# Supplementary material for: Dietary nutrients of relative importance associated with coronary artery disease: Public health implication from random forest analysis
Source: PLoS One. 2020 Dec 10;15(12):e0243063. doi: 10.1371/journal.pone.0243063 (PMC7728256; doi:10.1371/journal.pone.0243063)
Supplement: S1 Table — (DOCX) [file pone.0243063.s001.docx]

**S1 Table. List of food items commonly consumed by Nepalese in Nepal.**

| \| 1 \| **Drinking good items (5)** \| \| --- \| --- \| \| 1.1 \| Black tea with sugar \| \| 1.2 \| Coffee with sugar \| \| 1.3 \| Fruits juice \| \| 1.4 \| Soft Drinks (Coco cola, Fanta, Sprite, etc) \| \| 1.5 \| Alcohol (Wine, vodka, bear, whisky, ) \| \| **2** \| **Milk and milk products (5)** \| \| 2.1 \| Milk (whole/skim/powder) \| \| 2.2 \| Yogurt \| \| 2.3 \| Butter milk/ Lassi \| \| 2.4 \| Paneer/Cheese \| \| 2.5 \| Milk sweets \| \| **3** \| **Cereals product(16)** \| \| 3.1 \| White rice (Bhaat) \| \| 3.2 \| Beaten/puffed rice (Cheeura/Bhuja) \| \| 3.3 \| Corn porridge( Dhido) \| \| 3.4 \| Roasted Maize/grinned roasted maize flour (Saatu) \| \| 3.5 \| Boiled/barbequed green Maize \| \| 3.6 \| Millet local bread/porridge (Kodoko roti/Dhido) \| \| 3.7 \| Wheat local bread/Porridge ( Gilo roti/Dhido) \| \| 3.8 \| Wheat bread (Tawa/sukkah Roti) \| \| 3.9 \| Dough bread/toast \| \| 3.10 \| Pasta/Chawmen/Noodle \| \| 3.11 \| Samosha (Singhada) \| \| 3.12 \| Puri/Paratha \| \| 3.13 \| Jeri/sweets \| \| 3.14 \| Dalmod/Namkeen \| \| 3.15 \| MoMo (chicken/buffalo) \| \| 3.16 \| MoMo (Vegetables) \| \| 4 \| **Lentil/Pulses/beans (9)** \| \| 4.1 \| Lentils (Masuro daal) \| \| 4.2 \| Yellow gram (Arhar Daal) \| \| 4.3 \| Black Gram (Maas Daal \| \| 4.4 \| Mixed pulses (Mixed Daal) \| \| 4.5 \| Peas (Kerau Daal) \| \| 4.6 \| Rajmah/Beans curry \| | \| 4.7 \| Benghal gram (Channa) \| \| --- \| --- \| \| 4.8 \| Mixed beans/pulses (Quanti) \| \| 4.9 \| Roasted Soybean \| \| **5** \| **Egg/Meat/Fish (6)** \| \| 5.1 \| Boiled egg/ Umlet \| \| 5.2 \| Chicken meat \| \| 5.3 \| Goat meat \| \| 5.4 \| Buffalo meat \| \| 5.5 \| Pig meat \| \| 5.6 \| Fish \| \| 6 \| **Vegetables (5)** \| \| 6.1 \| Green leafy vegetables curry (Mustard leaf, Spinach, pumpkins leaf, fenugreek leaf, …) \| \| 6.2 \| Green Beans vegetables curry ( beans, long beans, green peas) \| \| 6.3 \| Other vegetables curry (Cauliflower, Cabbage, lady’s finger, pumpkin, guard ) \| \| 6.4 \| Potato cooked in curry \| \| 6.5 \| Salad (Cucumber, Onion, Radish , carrot, ) \| \| **7** \| **Fruits(8)** \| \| 7.1 \| Dry fruits (raisins and dates) \| \| 7.2 \| Dry fruits (nuts and others) \| \| 7.3 \| Banana \| \| 7.4 \| Apple \| \| 7.5 \| Mango \| \| 7.6 \| Orange \| \| 7.7 \| Pomegranate \| \| 7.8 \| Other seasonal fruits (Grapes. Papaya, Guava, Pine apple, Watermelon, Pear, lychee,) \| \| **8** \| **Fats/Oil(2)** \| \| 8.1 \| Animal butter/ghee/fat \| \| 8.2 \| Vegetables oil/butter/ghee \| \| 9 \| **Others food (3)** \| \| 9.1 \| Pizza \| \| 9.2 \| Burgers \| \| 9.3 \| Any type of chips \| |
| --- | --- | --- | --- | --- | --- | --- | --- | --- | --- | --- | --- | --- | --- | --- | --- | --- | --- | --- | --- | --- | --- | --- | --- | --- | --- | --- | --- | --- | --- | --- | --- | --- | --- | --- | --- | --- | --- | --- | --- | --- | --- | --- | --- | --- | --- | --- | --- | --- | --- | --- | --- | --- | --- | --- | --- | --- | --- | --- | --- | --- | --- | --- | --- | --- | --- | --- | --- | --- | --- | --- | --- | --- | --- | --- | --- | --- | --- | --- | --- | --- | --- | --- | --- | --- | --- | --- | --- | --- | --- | --- | --- | --- | --- | --- | --- | --- | --- | --- | --- | --- | --- | --- | --- | --- | --- | --- | --- | --- | --- | --- | --- | --- | --- | --- | --- | --- | --- | --- | --- | --- | --- | --- | --- | --- | --- | --- | --- | --- | --- | --- | --- | --- | --- | --- | --- | --- | --- |
