# Supplementary material for: Dietary nutrients of relative importance associated with coronary artery disease: Public health implication from random forest analysis
Source: PLoS One. 2020 Dec 10;15(12):e0243063. doi: 10.1371/journal.pone.0243063 (PMC7728256; doi:10.1371/journal.pone.0243063)
Supplement: S2 Table — ICC: Intraclass correlation coefficient; aData are shown as mean ± standard deviation (SD) (DOCX) [file pone.0243063.s002.docx]

**S2 Table. Mean daily nutrient intakes estimated by the average of three 24 h dietary recalls (DR) and two FFQ (food frequency questionnaire), and correlations between the two methods.**

|  |  |  |  | **Correlation** | | **Cross classification** | | |
| --- | --- | --- | --- | --- | --- | --- | --- | --- |
| **Nutrient** | **24h DR** | **FFQ1** | [**FFQ2**](mailto:FFQ@) | **DR and FFQ2** | **FFQ1**  **and FFQ2 (ICC)** | **% age in same quartiles** | **% age in extreme quartiles** | **Kappa coefficients** |
| **Energy (Kcal)** | 3013±667^a^ | 2998±468^a^ | 2970±445^a^ | 0.851 | 0.872 | 59 | 0 | 0.455 |
| **Carbohydrate (g)** | 501±138.6 | 490±102 | 474±71 | 0.753 | 0.874 | 53 | 0 | 0.374 |
| **Protein (g)** | 66.5±8.8 | 60.3±7.3 | 64.3±7.5 | 0.574 | 0.557 | 41.7 | 3.5 | 0.212 |
| **Fat/oil (g)** | 46.7±16 | 46.7±13.9 | 45.3±10.6 | 0.841 | 0.892 | 61.7 | 0 | 0.49 |
| **Cholesterol (mg)** | 182±78 | 174±63 | 172±59 | 0.453 | 0.747 | 41.7 | 4.3 | 0.223 |
| **Fiber (g)** | 7.7±2.8 | 7.1±2.9 | 7.1±2.8 | 0.463 | 0.749 | 30.4 | 4.3 | 0.072 |
| **Thiamine (mg)** | 1.9±0.8 | 2.2±0.5 | 1.9±0.8 | 0.492 | 0.55 | 47 | 2.6 | 0.293 |
| **Niacin (mg)** | 19.1±7.4 | 22±7.7 | 26.3±11.9 | 0.309 | 0.407 | 34.8 | 5.2 | 0.246 |
| **Riboflavin (mg)** | 1.3±0.4 | 1.3±0.3 | 1.3±0.3 | 0.548 | 0.57 | 49.6 | 4.3 | 0.327 |
| **Beta-carotene (mcg)** | 1414±788 | 1483±409 | 1476±473 | 0.207 | 0.622 | 39 | 5.2 | 0.189 |
| **Vitamin A (mcg)** | 441±178 | 453±128 | 434±172 | 0.6 | 0.747 | 43.5 | 0.9 | 0.246 |
| **Vitamin C (mg** | 61±17 | 59±17 | 61±12 | 0.453 | 0.62 | 35.6 | 4.3 | 0.142 |
| **Iron (mg)** | 11.4±2.6 | 11.3±3.2 | 11±2.6 | -0.003 | 0.256 | 25.2 | 13.9 | 0.003 |
| **Phosphorous (mg)** | 1574±440 | 1594±331 | 1589±248 | 0.797 | 0.883 | 53.9 | 0.9 | 0.385 |
| **Calcium (mg)** | 565±125 | 552±152 | 507±137 | 0.57 | 0.856 | 47.8 | 0.9 | 0.304 |
| **Zinc (mg)** | 10.5±2.9 | 10.6±4.3 | 10.6±4.3 | 0.516 | 0.749 | 37.4 | 3.5 | 0.165 |

ICC: Intraclass correlation coefficient

^a^Data are shown as mean ± standard deviation (SD)
