# Supplementary material for: Dietary nutrients of relative importance associated with coronary artery disease: Public health implication from random forest analysis
Source: PLoS One. 2020 Dec 10;15(12):e0243063. doi: 10.1371/journal.pone.0243063 (PMC7728256; doi:10.1371/journal.pone.0243063)
Supplement: S3 Table — kcal: kilocalorie; g: gram; mg: milligram; mcg: microgram; R.E.: retinol equivalent; PUFA: polyunsaturated fatty acid; MUFA: monounsaturated fatty acid; SFA: saturated fatty acid. aPaired t-test. bMean and standard deviation (SD) value. *p ≤ .05; **p≤ .01; ***p ≤ .001. (DOCX) [file pone.0243063.s003.docx]

**S3 Table. Distribution of nutritional factors associated with coronary artery disease between case and control groups in the study.**

| **Nutrients intake/day** | **Control**  **n=306** | **Case**  **n=306** | ***p*-value^a^** |
| --- | --- | --- | --- |
| **Food energy kcal** | 2716±394 ^b^ | 2702±423 | 0.655 |
| **Protein g** | 70±12 | 69±14 | 0.109 |
| **Total fat/oil g** | 55±12 | 63±15 | <0.001^***^ |
| **Carbohydrate g** | 439±73 | 420±73 | 0.001^***^ |
| **Fiber g** | 11.5±2.4 | 10.4±2.5 | <0.001^***^ |
| **Calcium mg** | 705±341 | 671±367 | 0.235 |
| **Phosphorus mg** | 1468±268 | 1432±304 | 0.117 |
| **Iron mg** | 21.8±5 | 21.2±6.6 | 0.155 |
| **Zinc mg** | 14.4±2.9 | 13.7±2.6 | 0.003^**^ |
| **Thiamine mg** | 1.2±0.27 | 1.17±0.28 | 0.009^**^ |
| **Riboflavin mg** | 1.12±0.33 | 1.06±0.34 | 0.013^*^ |
| **Niacin mg** | 14.7±4.09 | 14.7±4.4 | 0.919 |
| **Vitamin C mg** | 45.5±10.5 | 40.3±10.5 | <0.001^***^ |
| **β-carotene mcg** | 2697±764 | 2264±664 | <0.001^***^ |
| **Vitamin A R.E.** | 699±175 | 614±149 | <0.001^***^ |
| **PUFA g** | 18.7±7.3 | 19.9±8.3 | 0.039^*^ |
| **MUFA g** | 18±6.8 | 20.3±7.8 | <0.001^***^ |
| **SFA g** | 15.3±5.8 | 19.3±7.2 | <0.001^***^ |
| **Cholesterol mg** | 120±66 | 146±76 | <0.001^***^ |

kcal: kilocalorie; g: gram; mg: milligram; mcg: microgram; R.E.: retinol equivalent; PUFA: polyunsaturated fatty acid; MUFA: monounsaturated fatty acid; SFA: saturated fatty acid.

^a^Paired t-test.

^b^Mean and standard deviation (SD) value. ^*^*p* ≤ .05; ^**^*p*≤ .01; ^***^*p* ≤ .001
