# Supplementary material for: Dietary nutrients of relative importance associated with coronary artery disease: Public health implication from random forest analysis
Source: PLoS One. 2020 Dec 10;15(12):e0243063. doi: 10.1371/journal.pone.0243063 (PMC7728256; doi:10.1371/journal.pone.0243063)
Supplement: S4 Table — PUFA: polyunsaturated fatty acid; MUFA: monounsaturated fatty acid; SFA: saturated fatty acid. (DOCX) [file pone.0243063.s004.docx]

**S4 Table. Correlation matrix among eighteen dietary nutrients and energy intake.**

|  | **Food.energy** | **Carbohydrate** | **Protein** | **Total.fat.oil** | **PUFA** | **MUFA** | **SFA** | **Cholesterol** | **Fiber** | **Thiamine** | **Niacin** | **Riboflavin** | **Beta.carotene** | **Vitamin.A.R.E** | **Vitamin.C** | **Zinc** | **Iron** | **Calcium** | **Phosphorus** |
| --- | --- | --- | --- | --- | --- | --- | --- | --- | --- | --- | --- | --- | --- | --- | --- | --- | --- | --- | --- |
| **Food.energy** | 1 | 0.91 | 0.78 | 0.56 | 0.25 | 0.36 | 0.4 | 0.4 | 0.24 | 0.12 | 0.5 | 0.18 | 0.09 | 0.08 | 0.14 | 0.39 | 0.41 | 0.32 | 0.85 |
| **Carbohydrate** | 0.91 | 1 | 0.56 | 0.18 | 0.06 | 0.13 | 0.1 | 0.16 | 0.2 | 0.14 | 0.32 | 0.17 | 0.08 | 0.02 | 0.09 | 0.44 | 0.34 | 0.1 | 0.73 |
| **Protein** | 0.78 | 0.56 | 1 | 0.63 | 0.27 | 0.39 | 0.54 | 0.64 | 0.42 | 0.17 | 0.74 | 0.22 | 0.19 | 0.27 | 0.25 | 0.21 | 0.54 | 0.55 | 0.83 |
| **Total.fat.oil** | 0.56 | 0.18 | 0.63 | 1 | 0.49 | 0.63 | 0.75 | 0.57 | 0.06 | -0.03 | 0.43 | 0.06 | -0.02 | 0.1 | 0.09 | 0.07 | 0.21 | 0.5 | 0.53 |
| **PUFA** | 0.25 | 0.06 | 0.27 | 0.49 | 1 | -0.2 | 0.09 | 0.26 | 0.11 | -0.02 | 0.35 | -0.01 | -0.02 | 0.06 | 0.05 | 0.06 | 0.12 | -0.01 | 0.14 |
| **MUFA** | 0.36 | 0.13 | 0.39 | 0.63 | -0.2 | 1 | 0.4 | 0.33 | 0 | -0.01 | 0.29 | 0.08 | -0.01 | 0.02 | 0.03 | 0.04 | 0.15 | 0.29 | 0.32 |
| **SFA** | 0.4 | 0.1 | 0.54 | 0.75 | 0.09 | 0.4 | 1 | 0.48 | 0.05 | -0.01 | 0.16 | 0.06 | 0.02 | 0.13 | 0.1 | -0.01 | 0.14 | 0.73 | 0.53 |
| **Cholesterol** | 0.4 | 0.16 | 0.64 | 0.57 | 0.26 | 0.33 | 0.48 | 1 | 0.04 | 0.02 | 0.53 | 0.14 | 0.04 | 0.07 | 0.08 | 0.06 | 0.32 | 0.3 | 0.5 |
| **Fiber** | 0.24 | 0.2 | 0.42 | 0.06 | 0.11 | 0 | 0.05 | 0.04 | 1 | 0.21 | 0.37 | 0.05 | 0.26 | 0.53 | 0.43 | -0.04 | 0.49 | 0.18 | 0.19 |
| **Thiamine** | 0.12 | 0.14 | 0.17 | -0.03 | -0.02 | -0.01 | -0.01 | 0.02 | 0.21 | 1 | 0.12 | 0.65 | 0.02 | 0.03 | -0.03 | 0.02 | 0.11 | 0.05 | 0.1 |
| **Niacin** | 0.5 | 0.32 | 0.74 | 0.43 | 0.35 | 0.29 | 0.16 | 0.53 | 0.37 | 0.12 | 1 | 0.17 | 0.11 | 0.12 | 0.12 | 0.09 | 0.61 | -0.01 | 0.43 |
| **Riboflavin** | 0.18 | 0.17 | 0.22 | 0.06 | -0.01 | 0.08 | 0.06 | 0.14 | 0.05 | 0.65 | 0.17 | 1 | -0.07 | -0.06 | -0.09 | 0.07 | 0.07 | 0.07 | 0.17 |
| **Beta.carotene** | 0.09 | 0.08 | 0.19 | -0.02 | -0.02 | -0.01 | 0.02 | 0.04 | 0.26 | 0.02 | 0.11 | -0.07 | 1 | 0.4 | 0.4 | -0.06 | 0.14 | 0.17 | 0.15 |
| **Vitamin.A.R.E.** | 0.08 | 0.02 | 0.27 | 0.1 | 0.06 | 0.02 | 0.13 | 0.07 | 0.53 | 0.03 | 0.12 | -0.06 | 0.4 | 1 | 0.82 | -0.07 | 0.21 | 0.36 | 0.23 |
| **Vitamin.C** | 0.14 | 0.09 | 0.25 | 0.09 | 0.05 | 0.03 | 0.1 | 0.08 | 0.43 | -0.03 | 0.12 | -0.09 | 0.4 | 0.82 | 1 | -0.01 | 0.29 | 0.33 | 0.22 |
| **Zinc** | 0.39 | 0.44 | 0.21 | 0.07 | 0.06 | 0.04 | -0.01 | 0.06 | -0.04 | 0.02 | 0.09 | 0.07 | -0.06 | -0.07 | -0.01 | 1 | -0.03 | -0.02 | 0.33 |
| **Iron** | 0.41 | 0.34 | 0.54 | 0.21 | 0.12 | 0.15 | 0.14 | 0.32 | 0.49 | 0.11 | 0.61 | 0.07 | 0.14 | 0.21 | 0.29 | -0.03 | 1 | 0.18 | 0.39 |
| **Calcium** | 0.32 | 0.1 | 0.55 | 0.5 | -0.01 | 0.29 | 0.73 | 0.3 | 0.18 | 0.05 | -0.01 | 0.07 | 0.17 | 0.36 | 0.33 | -0.02 | 0.18 | 1 | 0.6 |
| **Phosphorus** | 0.85 | 0.73 | 0.83 | 0.53 | 0.14 | 0.32 | 0.53 | 0.5 | 0.19 | 0.1 | 0.43 | 0.17 | 0.15 | 0.23 | 0.22 | 0.33 | 0.39 | 0.6 | 1 |

PUFA: polyunsaturated fatty acid; MUFA: monounsaturated fatty acid; SFA: saturated fatty acid.
