# Supplementary material for: Dietary nutrients of relative importance associated with coronary artery disease: Public health implication from random forest analysis
Source: PLoS One. 2020 Dec 10;15(12):e0243063. doi: 10.1371/journal.pone.0243063 (PMC7728256; doi:10.1371/journal.pone.0243063)
Supplement: S1 File — (PDF) [file pone.0243063.s005.pdf]

मंजुरीपत्र

परिचयः म (तिल बहादुर बस्नेत) हाल महामारी रोग सम्बन्धी बिषयमा विध्यावारिधि तहको नान्जिंग मेडिकल विश्वबिध्यालय, चिनमा अध्ययनरत छु । मैले यस अध्ययन सम्बन्धी सुचनाहरु प्रदान गरेर तपाइलाई मेरो अध्ययनमा सहभागिताको लागि अनुरोध गर्नेछु । तपाइले अहिले नै निर्णय लिइहाल्नु पर्दैन र तपाइलाई लागेका अनुसन्धान सम्बन्धी कुराहरु सहजतरीकाले सोधपुछ गरी बुझ्न सक्नुहुनेछ । यदि तपाइले यस अध्ययनमा सहभागिता जनाउने निर्णय गर्नुभयो भने तपाइको सामाजिक तथा जानसान्खिक सूचना, धुम्रपान, मधेयपान तथा ब्यायम गर्ने बानीको साथै तपाइको दैनिक खाने खाना (पोषण) को बारेमा तपाइलाई सोध्नेछु साथै तपाइको रक्तचाप, तौल, उचाई तथा कम्मरको नाप लिइने छ । यसका लागि करिब ३०-४५ मिनेट समय लाग्नेछ ।

अनुसन्धानको उधेश्यः मुटुको रोग हाम्रो रहनसहन तथा आनिबानिसंग सम्बन्धित जटिल खालको रोग हो । पछिल्ला केहि बर्ष यता यो रोग दिन प्रतिदिन बढिरहेको छ । संसारभर यो रोग सम्बन्धी धेरै अनुसन्धान भएतापनि नेपाली समाजमा खासगरी यस रोगको सम्भावित जोखिम तत्वहरुको बारेमा धेरै कम अध्ययन भएको छ । त्यसैले यस अनुसन्धान ***"मुटुको धमनी सम्बन्धी रोग र पोषण तत्व खासगरी खानेतेल तथा घिऊ/ब्वोसो सम्बन्ध"*** कस्तो छ भन्ने बारेमा केन्द्रित हुनेछ ।

स्वेच्छिक सहभागिताः यस अनुसन्धानमा सहभागिता हुने वा नहुने भन्ने निर्णय तपाईको हो । तपाइले कुनै पनि बेला अन्तर्वातामा सहभागी नहुने भनि निर्णय लिन सक्नु हुनेछ ।

गोपनियताः यस अनुसन्धानमा प्राप्त भएका व्यक्तिगत सुचनाहरु गोप्य राखिने छ र यस अध्ययनको लागि मात्र प्रयोग गरिने छ साथै तपाईको नाम कतै काहिँ उल्लेख गरिने छैन ।

अनुसंधानको शारांश प्रस्तुतिः मैले यस अनुसन्धानको सारांश प्रकाशित गर्नेछु ताकी अरु मानिसले समेत यो कुरा जान्न पाउनेछन् ।

यो अनुसन्धानको प्रस्ताबना नेपाल स्वास्थ्य अनुसन्धान केन्द्रको इथिकल पुनारावलोकन समितिबाट स्वीकृत भैसकेको छ ।

स्वीकृत प्रमाणीकरण

मैले माथिका सबै सुचनाहरु पाँए र केहि प्रश्न गरी नबुझेको कुरा बुझ्ने मौका पनि पाँए । मैले मेरो राजीखुसीले यो अनुसन्धानको लागि मन्जुरी पत्र दिएको छु ।

सहभागीको नाम ....हस्ताक्षर .... मिति ....

यदि उत्तरदाता असाक्षर भएमा

मैले यो मंजुरिपत्रमा भएको सम्पूर्ण सुचनाहरु जानकारी दिएको र प्रश्न सोधी बुझ्ने मौका दिएको साथै स्वतस्फुत्र यो मन्जुरी नामा दिएको यकिन गर्दछु ।

साक्षीको नाम: सहभागीको नाम:

हस्ताक्षर............................... सहभागीको औठा छाप

मिति ....

उत्तरदाता संख्या .... मिति...................................

भाग क: मुटुको कोरोनरी रोगको लागि पोषणतत्व बाहेक अन्य जोखिम तत्वहरु

१. **बिगत कहिले देखि यो रोगको समस्या भएको हो (केसको लागि मात्र)**

....................दिन/महिना

२. **यो रोग कसरी सुरु भयो र कस्ता खालका लक्षणहरुदेखिए?..**

....

३. **के तपाइको परिवारका कुनै सदस्य साथै मामा घरमा, हजुरबुवा/आमालाई यस्तो खालको मुटु रोग थियो ?**

क.	हो १
ख.	होइन २
ग.	थाहछैन ७७

४. **तपाइलाई यो रोग बाहेक अन्य कुनै रोग छन या कुनै रोगको औषधि सेबन गर्नुहुन्छ**

क.	हो १
ख.	होइन २

यदि हो भने कुन कुन रोग हो/हुन् र कहिले देखि हो उल्लेख गर्नुहोस्...

रोगहरु	समय (.....बर्ष देखि)
उच्च रक्तचाप	
मधुमेह	
कोलेस्टेरोल घटबढीको समस्या	
थाईरोइडको समस्या	
अन्य........	

५. **के तपाई बिगत १ बर्ष देखि धुम्रपान गरिराक्नु भएको छ ?**

क.	हो १
ख.	होइन २

६. **यदि अहिले धुम्रपान गर्नुहुन्छ भने दिनमा कति खिल्ली चुरोट पिउनुहुन्छ ?**

....................खिल्ली

७. **यदि बिगतमा धुम्रपान गर्नुहुन्थ्यो भने दिनमा कतिवटा चुरोट पिउनुहुन्थ्यो ?**

..............................खिल्ली

८. **तपाइँ कति बर्षको हुदा चुरोट पिउन सुरुगर्नु भयो ?**

...........................बर्ष

९. **यदि पहिले पिउनुहुन्थ्यो र अहिले छोडेको हो भने कति समय सम्म चुरोट पिउनु भयो?**

....................दिन/महिना/बर्ष

१०. **तपाइले प्राय कुन प्रकारको चुरोट पिउनुहुन्छ ?**

क.	उत्पादित चुरोट (सिखर, खुकुरी, सुर्य)..======..१
ख.	हातले बेरेको चुरोट (बिडी)..................२
ग.	अन्य उल्लेख..................................८८

११. **तपाइको घरपरिवारमा अरु सदस्यहरु कसैले तपाइको उपस्तिथीमा चुरोट पिउनेगर्नुहुन्छ ?**

क.	हो १
ख.	होइन २
ग.	कहिलेकाही ३

१२. **के तपाई बिगत १ बर्ष देखि दैनिक सुर्तीजन्य बस्तु जस्तै पान, खैनी, सुर्ती, गुड्का) सेबन गर्नुहुन्छ?**

क.	हो १
ख.	होइन २
ग.	कहिलेकाही ३

१३. **के तपाई बिगतमा दैनिक सुर्तीजन्य बस्तु जस्तै पान, खैनी, सुर्ती, गुड्का) सेबन गर्नुहुन्थ्यो?**

क.	हो १
ख.	होइन २

१४. **के तपाई बिगत १ बर्ष देखि मध्यपान गरिराख्नुभएको छ ? यदि गर्नुहुन्न भने प्रश्न १७ मा जानुहोस्**

क.	हो १
ख.	होइन २
ग.	पहिले गर्थे अहिले छैन......................३

१५. **बिगत १ बर्ष देखि कम्तिमा एउटा मध्यपान कत्तिको ध्यपान गर्नुहुन्छ ?**

क.	दैनिक १
ख.	हप्ताको ५-६ दिन २
ग.	हप्ताको १-४ दिन ३
घ.	महिनाको १-३ दिन ४
ङ.	महिनाको १ दिन भन्दा कम ५

१६. **बिगत ३० दिन भित्र तपाइले सालाखाला एक पटक मध्यपान गर्दा कति प्याक रक्सि सेवन गर्नुभयो ?**

................................प्याक

**Types of alcohol**	**Concentration of alcohol**	**1 standard drink**
Beer, Jaand and Tongba	5%	250 ml
Local raksi	27%	45 ml
Whisky, Vodka (spirits), Rum	40%	30 ml
Wine (red and white)	12%	105 ml

१७. **के तपाइको काम कम्तिमा १० मिनेट सम्म लगातार एकदमै कडा परिश्रम चाहिने जसले गर्दा धेरै पटक श्वासप्रस्वास भै मुटुको धड्कन बढ्ने खालको (जस्तै: ग्रहुंगो समान उठाने खालको, खन्ने तथा कन्स्ट्रक्सनको काम) छ ?**

२६. **के तपाले कम्तिमा ३० मिनेट सम्म लगातार मध्यम परिश्रम चाहिने जसले गर्दा श्वासप्रस्वास तथा मुटुको धड्कन बढ्ने खालको शारीरिक ब्यायाम तथा यात्रा (जस्तै: पौडी खेल्ने, ब्याडमिन्टन, टेबल टेनिस खेल्ने, योगा गर्ने, घर बाट बस चढ्न पैदल हिड्ने, हिडेरै कार्यालय जाने) गर्नुहुन्छ**

क.	हो १	
ख.	होइन २	

१८. एक हप्तामा कति दिन यस्तो एकदमै कडा परिश्रम चाहिने कार्य गर्नुहुन्छ ?

.......................दिन

१९. एक दिनमा कति घण्टा यस्तो मध्यम परिश्रम चाहिने कार्य गर्नुहुन्छ ?

..................घण्टा

२०. के तपाइको काम कम्तिमा ३० मिनेट सम्म लगातार मध्यम परिश्रम चाहिने र श्वासप्रस्वास तथा मुटुको धड्कन बढ्ने खालको (जस्तैः हल्का समान उठाने खालको, हिड्ने, कपडा धुने, कोठा सफा गर्ने, पौडी खेल्ने, ब्याडमिन्टन, टेबल टेनिस खेल्ने, योगा गर्ने) छ ?

क.	हो १
ख.	होइन २

२१. एक हप्तामा कति दिन यस्तो मध्यम परिश्रम चाहिने कार्य गर्नुहुन्छ ?

.......................दिन

२२. एक दिनमा कति घण्टा यस्तो मध्यम परिश्रम चाहिने कार्य गर्नुहुन्छ ?

..................घण्टा

२३. के तपाले कम्तिमा १० मिनेट सम्म लगातार एकदमै कडा परिश्रम चाहिने जसले गर्दा धेरै पटक श्वासप्रस्वास भै मुटुको धड्कन बढ्ने खालको शारीरिक ब्यायाम (जस्तैः बास्केट बल तथा फुटबल खेल्ने, छिटो छिटो बाईसीकल चलाउने, पौडी खेल्ने, दगुर्ने) गर्नुहुन्छ ?

क.	हो १
ख.	होइन २

२४. एक हप्तामा कति दिन यस्तो कडा परिश्रम चाहिने खालको शारीरिक ब्यायाम गर्नुहुन्छ ?

................दिन

२५. एक दिनमा कति घण्टा यस्तो कडा परिश्रम चाहिने खालको शारीरिक ब्यायाम गर्नुहुन्छ ?

................घण्टा

क.	हो १
ख.	होइन २

२७. एक हप्तामा कति दिन यस्तो मध्यम परिश्रम चाहिने शारीरिक ब्यायाम तथा यात्रा गर्नुहुन्छ

.......................दिन

२८. एक दिनमा कति घण्टा यस्तो मध्यम परिश्रम चाहिने शारीरिक ब्यायाम तथा यात्रा गर्नुहुन्छ ?

....................घण्टा

२९. सुत्ने बाहेक तपाइले कति समय बसेर वा कुनै काम नगरी (पढने, बस तथा मोटरसीकलमा यात्रा गर्ने,साथीहरु संग बस्ने, टेलिभिजन हेर्ने) बिताउनुहुन्छ ?

.....................घण्टा

३०. तपाई सरदरमा कति घण्टा सुत्नुहुन्छ ?

क.	४ घण्टा भन्दा कम..........................१
ख.	४-६ घण्टा २
ग.	६-८ घण्टा ३
घ.	८-१० घण्टा ४
ङ	१० घण्टा भन्दा धेरै..........................५

३१. के तपाई रातिमा काम गर्नुपर्ने खालको पेशा गर्नुहुन्थ्यो/गर्नुहुन्छ?

क.	हो १
ख.	होइन २

३२ के तपाइलाई मानसिक रोग सम्बन्धी कुनै समस्या छ या सो रोगको औषधि शेबन गर्नुहुन्छ?

क.	हो १
ख.	होइन २

३३. यदि मानसिक रोग सम्बन्धी समस्या छ भने कहिले देखी भयो ?

क.	........................दिन/महिना/बर्ष
ख.	थाहछैन ७७

भाग ख: खानाको पटक जांच प्रश्नावली

यदि तपाइले बिगत एक बर्ष भित्र यी तलका खाना खानुभयो भने सबैभन्दा उपयुक्त छनौट गर्नुहोस्।

खानेकुराको प्रकार	इकाई	चित्र नम्बर/ खानाको आकार	कहिल्यै पनि वा < महिनाको एकपटक	१-३ पटक / महिना	हप्ताको १ पटक	२-४ पटक/ हप्ता	५-६ पटक / हप्ता	१ पटक/ दिन	२-३ पटक / दिन	४-५ पटक / दिन	६+ पटक / दिन
१) पेय पदार्थ (५)											
चिनी राखे/ नराखेको कालो चिया			१	२	३	४	५	६	७	८	९
चिनी राखे/ नराखेको दुध चिया			१	२	३	४	५	६	७	८	९

चिनी राखे/ नराखेको कफी			१	२	३	४	५	६	७	८	९
फलफूलको जुस			१	२	३	४	५	६	७	८	९
हल्का पेय पदार्थ (कोक/फ्यान्टा/स्पराइट)			१	२	३	४	५	६	७	८	९
अन्य.......			१	२	३	४	५	६	७	८	९
**२) दुध तथा दुध जन्य पदार्थ (५)**											
दुध<br>(पूर्ण/ स्किम (तर निकालेको)/पाउडर)			१	२	३	४	५	६	७	८	९
दही			१	२	३	४	५	६	७	८	९
मही, लस्सी			१	२	३	४	५	६	७	८	९
पनीर, चिज			१	२	३	४	५	६	७	८	९
दुधबाट बनेको मिठाई			१	२	३	४	५	६	७	८	९
अन्य....			१	२	३	४	५	६	७	८	९
**कहिले देखी दुध** दही **पिउदै हुनुहुन्छ** क.........................बर्ष..........................१ ख. थाहाछैन ७७											
**३. अन्न (१५)**											
चामलको भात			१	२	३	४	५	६	७	८	९
चिउरा, भुजा			१	२	३	४	५	६	७	८	९
मकै (च्याख्ला, ढिडो)			१	२	३	४	५	६	७	८	९
उसिनेको तथा पोलेको मकैको घोगा (सिजनमा)			१	२	३	४	५	६	७	८	९
भुटेको मकै/सातु/ कर्नफ्लेक्स,			१	२	३	४	५	६	७	८	९
ओट/मुसेली (Oat)			१	२	३	४	५	६	७	८	९
गहुको (whole grain) पिठो बाट बनेको सुख्खा रोटि तथा रोटि			१	२	३	४	५	६	७	८	९
कोदो/गहुको ढिडो			१	२	३	४	५	६	७	८	९
पास्ता/मेक्रोनी/चाउमिन/नुडल/चाउचाउ			१	२	३	४	५	६	७	८	९
भेज/चिकेन/बफ म:म:			१	२	३	४	५	६	७	८	९
ब्रेअड/ टोस्ट/पाउरोटी			१	२	३	४	५	६	७	८	९
बिस्कुट/केक/कुक्किज			१	२	३	४	५	६	७	८	९
समोसा/सिंगड़ा			१	२	३	४	५	६	७	८	९
जेरी-पुरी			१	२	३	४	५	६	७	८	९
दालमोड			१	२	३	४	५	६	७	८	९
अन्य			१	२	३	४	५	६	७	८	९
अन्न सम्बन्धी पूर्ण सूचना पाउनको लागि ***तपाइले बिहान दिउसो खाजा नास्ता के के खानु हुन्छ*** भनेर सोध्नुहोस?											
**४) दाल तथा सिमि/बोडी (११)**											
**कुनै प्रकारको दाल तथा सिमि/बोडी**			१	२	३	४	५	६	७	८	९
**दाल तथा सिमि/बोडी कत्तिको बाक्लो खानुहुन्छ क. धेरै बाक्लो (कचौरा बाट झर्ने गारो हुने)...........२ ख. ठिकै बाक्लो (कचौरा ख बाट सजिलै झर्ने) ....३ ग. पातलो (भातको माड जस्तो)...................३**											
रहर दाल			१	२	३	४	५	६	७	८	९
मुंग दाल			१	२	३	४	५	६	७	८	९
**खानेकुराको प्रकार**	**इकाई**	**चित्र नम्बर/ खानाको आकार**	**कहिल्यै पनि वा < महिनाको एकपटक**	**१-३ पटक / महिना**	**हप्ताको १ पटक**	**२-४ पटक/ हप्ता**	**५-६ पटक / हप्ता**	**१ पटक/ दिन**	**२-३ पटक / दिन**	**४-५ पटक / दिन**	**६+ पटक / दिन**
मास दाल			१	२	३	४	५	६	७	८	९
मसुरो दाल			१	२	३	४	५	६	७	८	९
चनाको दाल			१	२	३	४	५	६	७	८	९
मिश्रण दाल			१	२	३	४	५	६	७	८	९
गेडागुडीको दाल(क्वाटी)			१	२	३	४	५	६	७	८	९
केराउको, राजमहाको दाल			१	२	३	४	५	६	७	८	९
चंना उसिनेको			१	२	३	४	५	६	७	८	९
भुटेको भटमास			१	२	३	४	५	६	७	८	९
अन्य ..			१	२	३	४	५	६	७	८	९

**५) अन्डा, माछा मासु (९)**											
कुखुराको उमालेको अन्डा, अम्लेट, तरकारी			१	२	३	४	५	६	७	८	९
कुखुराको मासु तेल/ घिऊमा तारेको/भुटेको			१	२	३	४	५	६	७	८	९
कुखुराको मासु ग्रेभी			१	२	३	४	५	६	७	८	९
बोकाखसीको मासु ग्रेभी			१	२	३	४	५	६	७	८	९
राँगाको मासु तेल/ घिऊमा तारेको भुटेको			१	२	३	४	५	६	७	८	९
राँगाको मासुको साँधेको छोइला बचेला सुकुटी			१	२	३	४	५	६	७	८	९
राँगाको मासु ग्रेभी			१	२	३	४	५	६	७	८	९
बंगुरको मासु तेल/ घिऊमा तारेको भुटेको ग्रेभी			१	२	३	४	५	६	७	८	९
माछा तेल/ घिऊमा तारेको भुटेको, ग्रेभी			१	२	३	४	५	६	७	८	९
अन्य			१	२	३	४	५	६	७	८	९
**६) सुक्खा फलफूलहरु (७)**											
मम्फुली/बदाम (**peanut**)			१	२	३	४	५	६	७	८	९
आल्मोंड, काजु, किशमिश,			१	२	३	४	५	६	७	८	९
छुहारा/डेट्स/खजुर, पिस्ता, ओखर			१	२	३	४	५	६	७	८	९
अन्य			१	२	३	४	५	६	७	८	९
**७) साग तरकारी (९+१९=२८)**											
**तपाइले कुनै प्रकारको साग कत्तिको खानुहुन्छ?**					क.	बर्ष भरि नै		ख.	सिजनमा मात्र		
कुनै प्रकारको साग (बर्ष भरिको लागि)			१	२	३	४	५	६	७	८	९
कुनै प्रकारको साग (सिजन भरिको लागि)			१	२	३	४	५	६	७	८	९
रायो/ तोरीको साग			१	२	३	४	५	६	७	८	९
पालुन्गो/चम्सुरको साग			१	२	३	४	५	६	७	८	९
लट्टे(लोडे)को साग			१	२	३	४	५	६	७	८	९
मेथीको, बेथेको साग			१	२	३	४	५	६	७	८	९
फर्सीको मुन्टाको साग			१	२	३	४	५	६	७	८	९
कर्कोलोको, निउरोको साग			१	२	३	४	५	६	७	८	९
अन्य साग.....			१	२	३	४	५	६	७	८	९
**तरकारी कुनै प्रकारको**			१	२	३	४	५	६	७	८	९
**के तपाइले साग र माछा मासु तथा दाल पकाउदा पनि तरकारी पकाउनु हुन्छ ? क. हो ख. होइन**											
बन्दागोबी, काउली			१	२	३	४	५	६	७	८	९
ब्रोकौली, च्याउ			१	२	३	४	५	६	७	८	९
हरियो/पहेलो फर्सी			१	२	३	४	५	६	७	८	९
सिमि/बोडीको तरकारी			१	२	३	४	५	६	७	८	९
भिंडी/ पर्बर			१	२	३	४	५	६	७	८	९
**खानेकुराको प्रकार**	**इकाई**	**चित्र नम्बर/ खानाको आकार**	**कहिल्यै पनि वा < महिनाको एकपटक**	**१-३ पटक / महिना**	**हप्ताको १ पटक**	**२-४ पटक/ हप्ता**	**५-६ पटक / हप्ता**	**१ पटक/ दिन**	**२-३ पटक / दिन**	**४-५ पटक / दिन**	**६+ पटक / दिन**
तिते करेला			१	२	३	४	५	६	७	८	९
भान्टाको तरकारी			१	२	३	४	५	६	७	८	९
लौका/घिरौलाको तरकारी			१	२	३	४	५	६	७	८	९
स्कुसको तरकारी			१	२	३	४	५	६	७	८	९
भेडे खुर्सानी (Capsicum)			१	२	३	४	५	६	७	८	९
टमाटर			१	२	३	४	५	६	७	८	९
आलु			१	२	३	४	५	६	७	८	९
गाजर/काक्रो/मुला/कागती			१	२	३	४	५	६	७	८	९
अन्य			१	२	३	४	५	६	७	८	९
**मसलाजन्य खानेकुरा (६)**											
हरियो/ सुकेको खुर्सानी, लसुनको पोटि/कोसा,			१	२	३	४	५	६	७	८	९

अदुवा, प्याज											
धनिया/जिरा/करी मसला/ बेसार			१	२	३	४	५	६	७	८	९
**८) फलफूलहरु (१३)**											
**फलफूलहरु (कुनै प्रकारको)**			१	२	३	४	५	६	७	८	९
केरा			१	२	३	४	५	६	७	८	९
स्याउ			१	२	३	४	५	६	७	८	९
**नोट: अनार सुन्तला/ जुनार आँप, अंगुर को लागि सिजनको बेलामा महिना वा हप्ताको कतिपटक खानुहुन्छ भनेर सोध्नुहोस ।**											
अनार			१	२	३	४	५	६	७	८	९
सुन्तला/ जुनार			१	२	३	४	५	६	७	८	९
आँप			१	२	३	४	५	६	७	८	९
**नोट: नासपाती, अम्बा, भुइकटहर्, मेवा, खर्भुजा, लिच्ची, स्ट्रबेरी को लागि सिजनको बेलामा महिना वा हप्ताको कतिपटक खानुहुन्छ भनेर सोध्नुहोस।**											
अंगुर			१	२	३	४	५	६	७	८	९
नासपाती/अम्बा/ मेवा,			१	२	३	४	५	६	७	८	९
भुइकटहर्/खर्भुजा/लिच्ची			१	२	३	४	५	६	७	८	९
अन्य			१	२	३	४	५	६	७	८	९
**९) अन्य खाना (५)**			१	२	३	४	५	६	७	८	९
आलु चिप्स/अन्य चिप्स			१	२	३	४	५	६	७	८	९
पिज्जा/बर्गर/स्याण्डविच			१	२	३	४	५	६	७	८	९
अन्य			१	२	३	४	५	६	७	८	९
**१०) घिऊ/नौनी तथा ब्वोसो (४) चिया चमच**											
बनस्पति घिऊ/नौनी			१	२	३	४	५	६	७	८	९
जनावर (गाई/ भैसी/बाख्रा) को घिऊ/ नौनी			१	२	३	४	५	६	७	८	९
जनावर(बाख्रा/ बंगुर) को ब्वोसो			१	२	३	४	५	६	७	८	९
अन्य			१	२	३	४	५	६	७	८	९

अन्य खानेकुरा हप्ताको १ पटक भन्दा बडी खाने गर्नुभएको छ ? क. हो ख. होइन

	**खानेकुरा**	**प्रायखाने खानाको आकार**	**प्रत्येक हप्ता खानाको पटक**

भाग ग: तेल तथा घिऊ खपत सम्बन्धी प्रश्नावली

१ खाना पकाउनको लागि प्रायजसो कुन खानेतेल वा घिउ/ब्वोसो प्रयोग गर्नुहुन्छ ?

क	तोरीको तेल १
ख	सूर्यमुखी तेल २
ग	भटमासको तेल ३
घ	बनस्पति घिऊ/नौनी ४
ङ	जनावर (गाई, भैसी) को घिऊ/नौनी ५
च	जनावर(बाख्रा, बंगुर) को ब्वोसो.................६
छ	अन्य उल्लेखगर्नुहोस.................................८८

२ कहिले देखी यो खानेतेल प्रयोग गर्दै हुनुहुन्छ ?

क	.........................बर्ष...........................१
ख	थाहाछैन ७७

३. के तपाइले माथि उल्लेखित विभिन्न खाले खानेतेल फेरबदल गरी प्रयोग गर्नुहुन्छ ?

क	हो १
ख	होइन २
ग	कहिलेकाही ३
घ	थाहाछैन ७७

४. यदि फेरबदल गरी प्रयोग गर्नुहुन्छ भने

क	प्राय तोरी र कहिलेकाही अन्य.......................१
ख	प्राय सूर्यमुखी र कहिलेकाही अन्य................२
ग	प्राय बनस्पति घिऊ र कहिलेकाही अन्य.........३
घ	अन्य भए उल्लेखगर्नुहोस...........................८८

५. यसोगरी कहिले देखी खानेतेल प्रयोग गर्दै हुनुहुन्छ ?

क	.........................बर्ष...........................१
ख	थाहाछैन ७७

६. पहिले भन्दा अहिले तेलको प्रयोग खानेकुरामा कस्तो छ ?

क	बढेको १
ख	घटेको २
ग	कुनै परिबर्तन छैन ३
घ	थाहाछैन ७७

७. पहिले भन्दा अहिले तेलको प्रयोग बढाएको वा घटाएको हो भने कहिले देखि यसो गर्दै हुनुहुन्छ ?

क	.........................बर्ष...........................१
ख	थाहाछैन ७७

८. यदि तपाई १ लिटर खानेतेल किन्नुभयो भने तपाइको परिवारलाई सरदरमा कति दिनलाई पकाउन पुग्छ ?

क	......................दिन
ख	थाहाछैन.....................................७७

सालाखाला १ दिनमा तपाइको परिवारलाई खान पकाउन कति तेल खपत गर्नुहुन्छ ? (१ मुठी=५० मि.लि., १ देउवा/चौथाई=१२५ मि.लि., ५ मुठी=२५० मि.लि., १ माना=५०० मि.लि.)

.................मि. लि.

९. तपाइको परिवारमा कति जना संगै पकाएर खानुहुन्छ ?

< ३ बर्ष..........जना......१ ३-५ बर्ष...........जना.....२

५-७ बर्ष..........जना.......३ ७-९ बर्ष.........जना......४

९-१२ बर्ष..........जना.....५ १२–२१ बर्ष.......जना....६

>२१ बर्ष....................जना.............................७

१०. के तपाई घिऊ खाने गर्नुहुन्छ ?

क	हो १
ख	होइन २
ग	कहिलेकाही ३
घ	पहिले खान्थे अहिले खादिन ४

११. यदि खानुहुन्छ भने कुन घिऊ खाने गर्नुहुन्छ ?

क	नौनी घिऊ १
ख	खारेको घिऊ २
ग	बनस्पति घिऊ(**Peanut Butter**) ३
घ	अन्य भए उल्लेख गर्नुहोस........................६६

१२. कहिले देखी घिऊ खाने गर्नुभएको हो ?

क	.......................बर्ष.........................१
ख	थाहाछैन ७७

१३. यदि तपाई मासुमा बोसो देख्नु भयो भने के गर्नु हुन्छ

क	देखेजति फ्याक्ने १
ख	केहि नगरी खाने २
ग	मासुलाई पहिले उमाल्ने अनि पकाएर खाने..३

१४. तपाई सेकुवा तथा सुकुटी मासु कत्तिको खानु हुन्छ ?

क	कहिलेपनि खादिन...................................१
ख	हप्ताको एक पटक भन्दा कम..................२
ग	हप्ताको १-३ पटक ३
घ	हप्ताको ४-६ पटक ४
ङ	प्रतेक दिन ५

१५. घरमा / घर बाहिर धेरैभुटेको (deep fry) तथा तारेको खानेकुरा कत्तिको खानु हुन्छ ?

१६. यदि धेरैभुटेको (deep fry) तथा तारेको खानेकुरा खानुहुन्छ भने कहिले देखि खादै हुनु हुन्छ ?

क	कहिलेपनि खादिन..................................१
ख	हप्ताको एक पटक भन्दा कम................२
ग	हप्ताको १-३ पटक ३
घ	हप्ताको ४-६ पटक ४
ङ	प्रतेक दिन ५

क	..........................बर्ष.............................१
ख	थाहाछैन ७७

१७. तपाईलाई तेल/घिऊ/ब्वोसोयुक्त, तरयुक्त चिल्लो, भुटेको, तारेको खानेकुरा,अन्डाको पहेलो भाग खान मन पर्छ

धेरै नै ....५ धेरै ....४ ठिकै................३

अलिकम ....२ धेरै कम १

नाम :... ठेगाना.................................. सम्पर्क न.:.......................................

घ.व्यक्तिगत विवरण

१. थर/जातिः

क.	ब्राह्मण/छेत्री/ठकुरी	१
ख.	नेवार	२
ग.	जनजाती	३
घ.	दलित	४
ङ.	तराई मधेश जाति.............................	५
च	अन्य	८८

२ उमेरः. .... बर्ष

३. लिंगः

क.	महिला	१
ख.	पुरुष	२
ग.	तेस्रो लिंगी	३

४. बैबाहिक स्तिथिः

क.	हाल सम्म अबिबाहित	१
ख.	बिबाहित संगै बसेको/ नछुट्टीएको.......	२
ग.	सम्बन्ध बिच्छेद /छुट्टीएर बसेको.......	३
घ.	श्रीमान/श्रीमती एकको मृतु................	४

५. पेशाः

क.	घरको काम	१
ख.	कृषि	२
ग.	नोकरी/ शिक्षण	३
घ.	आफ्नैं काम/ सानो ब्यवशाय	४
ङ.	ठुलो ब्यबशाय (>१५ कर्मचारी).............	५
च.	अवकास	६
छ.	केहि छैन	७

६. शैक्षिक स्तर

क.	निरक्षर	१
ख.	सामान्य लेखपढ/प्राथमिक	२
ग.	निम्न माध्यमिक	३
घ.	माध्यमिक	४
ङ.	उच्च माध्यमिक	५
च	स्नातक वा माथि	६

७. परिवारको मासिक आम्दानी

क.	<१००००	१
ख.	१००००-२००००	२
ग.	२००००-३००००	३
घ.	३००००-४००००	४
ङ.	४०००० - ५००००	५
छ	>५००००	६
ज	थाहाछैन ७७	

भाग: ग **Anthropometric and other measurement**

1	उचाई Height cm		8	Blood sugar	
2	तौल Weight kg		9	Total cholesterol	
3	BMI		10	Triglycerides	
4	Waist cm		11	HDL level	
5	Hip cm		12	LDL level	
6	Waist and Hip ratio		13	Blood grouping	
7	Blood pressure	........................mm of hg	14	CPK MB	
