# Supplementary material for: Dietary nutrients of relative importance associated with coronary artery disease: Public health implication from random forest analysis
Source: PLoS One. 2020 Dec 10;15(12):e0243063. doi: 10.1371/journal.pone.0243063 (PMC7728256; doi:10.1371/journal.pone.0243063)
Supplement: S2 File — (PDF) [file pone.0243063.s006.pdf]

Consent form for hospital based case control study

[Informed Consent form for coronary heart disease patients (Cases), and stress exercise test negative and normal angiography (Control)]

The title of our research project: *"Nutritional factors special focus on dietary fat and oil in association with coronary heart diseases in Nepal: A hospital based case control study"*

[Name of Principal Investigator: Til Bahadur Basnet]

[Name of Organization: Nanjing Medical University, Little Buddha College of Health Science]

PART I: Information Sheet

Introduction

I am Til Bahadur Basnet, studying doctoral degree in Nanjing Medical University. We are doing research on coronary heart disease (CAD). I am going to give you information and invite you to be part of this research. Before you decide, you can talk to anyone you feel comfortable with about the research. There may be some words that you do not understand. Please ask me to stop as we go through the information and I will take time to explain. If you have questions later, you can ask them of me. If you decide to participate, we will ask some questions related to your socio demographic information, smoking, alcohol drinking habit and exercises and also diet habit. The whole process takes around 30-45 minutes.

Purpose of the research

Heart disease is one of the most common and dangerous diseases in this region. Although a lot of research has been conducted all over the word, information regarding the risk factors for the disease in our population is inadequate. The present research is focused on risk factors of CAD special focus on dietary factors.

Voluntary Participation

Your participation in this research is entirely voluntary. It is your choice whether to participate or not. Whether you choose to participate or not, all the services you receive at this clinic will

continue and nothing will change. You may change your mind later and stop participating even if you agreed earlier.

Confidentiality

We will not be sharing the identity of those participating in the research. The information that we collect from this research project will be kept confidential. Information about you that will be collected during the research will be put away and no-one but the researchers will be able to see it. Any information about you will have a number on it instead of your name. Only the researchers will know what your number is and we will lock that information up with a lock and key.

Sharing the Results

Confidential information will not be shared. We will publish the results in order that other interested people may learn from our research.

Right to Refuse or Withdraw

You do not have to take part in this research if you do not wish to do so and refusing to participate will not affect your treatment at this clinic in any way. You may stop participating in the research at any time that you wish without losing any of your rights as a patient here. Your treatment at this clinic will not be affected in any way.

This proposal has been reviewed and approved by the Ethics Review Committee of Nepal Health Research Council.

PART II: Certificate of Consent

I have read the foregoing information, or it has been read to me. I have had the opportunity to ask questions about it and any questions that I have asked have been answered to my satisfaction. I consent voluntarily to participate as a participant in this research.

Name of Participant___________________

Signature of Participant ____________________

Date ____________________________

Day/month/year

If illiterate

A literate witness must sign (if possible, this person should be selected by the participant and should have no connection to the research team). Participants who are illiterate should include their thumb-print as well.

I have witnessed the accurate reading of the consent form to the potential participant, and the individual has had the opportunity to ask questions. I confirm that the individual has given consent freely.

Name of witness______________________ AND Thumb print of participant

Signature of witness _______________________ Date _________________________

Day/month/year

Statement by the researcher/person taking consent

I have accurately read out the information sheet to the potential participant.

I confirm that the participant was given an opportunity to ask questions about the study, and all the questions asked by the participant have been answered correctly and to the best of my ability.

I confirm that the individual has not been coerced into giving consent, and the consent has been given freely and voluntarily.

A copy of this ICF has been provided to the participant.

Name of Researcher/person taking the consent_________________________

Signature of Researcher /person taking the consent___________________________

Date ____________________________

Day/month/year

Investigator's signature:…………………………………...

Date: Date ____________________________

Day/month/year
