# Supplementary material for: Dietary nutrients of relative importance associated with coronary artery disease: Public health implication from random forest analysis
Source: PLoS One. 2020 Dec 10;15(12):e0243063. doi: 10.1371/journal.pone.0243063 (PMC7728256; doi:10.1371/journal.pone.0243063)
Supplement: S3 File — (PDF) [file pone.0243063.s007.pdf]

Dietary survey questionnaire

Respondents No .... **Date..................................**

Section A: Cardiometabolic and behavioral risk factors related questionnaire

1. How many days do you have this problem? **(**FOR CASE ONLY **)**
   ....Days/months
2. Do you have this problem in your family members (maernal grand parents/maternal uncle/paternal uncle/aunt**?**

A.	No 1
B.	Yes 2
C.	Don't know 77

3. Do you have any other diseases or taking medicines for others diseases than this one?

A.	No 1
B.	Yes 2

4. if yes, please state which kind of diseases and for how many years?..

Disease	Duration (…………)
Blood pressure	
Diabetes	
Dyslipidemia	
Yhyroid problem	
Others.......	

5. Do you currently smoke any tobacco products, such as cigarettes, cigars, pipes, bidis, hukahs or tamakhus daily?

A.	No 1
B.	Yes 2

6. If yes, How many stick do you smoke the tobacco product per day **?**
   ....sticks
7. Did you smoke tobacco products in the past daily?

A.	No 1
B.	Yes 2

8. If yes, How many stick did you smoke the tobacco product per day**?**
   .... sticks
9. How old were you when you first started smoking?
   ....years
10. If you used to smoke in the past and now quited, how long did you smoke the tobacco product**?**
    ....days/months/years
11. Which type of cigarette do you/ used to smoke usualy**?**

A.	Manufactured (Shikher/Surya/Khukuri)….1
B.	Hand made (Bidi)......................................2
C.	Others (please state)..............................88

12. During the past 7 days, on how many days did someone in your home smoke when you were present?

A.	No 1
B.	Yes 2
C.	Sometimes 3

13. Do you currently use any smokeless tobacco products such as *[snuff, chewing tobacco, nasal snuff, khaini, surti, gutka] daily*?

A.	No 1
B.	Yes 2
C.	Sometimes 3

14. How old were you when you first started smoking?
    ....years
15. Did you use smokeless tobacco products in the past daily?

A.	No 1
B.	Yes 2

16. If you used to use smokeless tobacco in the past and now quited, how long did you smoke the tobacco product**?**
    ....days/months/years
17. Have you consumed an alcoholic drink within the past 12 months?

A.	No 1
B.	Yes 2

18. During the past 12 months, how frequently have you had at least one alcoholic drink?

A.	Daily 1
B.	5–6 days per week 2
C.	1–4 days per week 3
D.	1–3 days per month 4
E.	Less than once a month 5

19. During the past 30 days, when you drank alcohol, on average, how many occasion and standard alcoholic drinks per occasion did you have during one drinking occasion?
    ....Occasion............Pack/occasion

Types of alcohol	Concentration of alcohol	1 standard drink
Beer, Jaand and Tongba	5%	250 ml
Local raksi	27%	45 ml
Whisky, Vodka (spirits), Rum	40%	30 ml
Wine (red and white)	12%	105 ml

20. Does your work involve vigorous-intensity activity that causes large increases in breathing or heart rate *[carrying or lifting heavy loads, digging or construction work]* for at least 10 minutes continuously?

A.	No 1
B.	Yes 2

21. In a typical week, on how many days do you do vigorous-intensity activities as part of your work?

........................days

22. How much time do you spend doing vigorous-intensity activities at work on a typical day?

...................hours: minutes

23. Does your work involve moderate-intensity activity that causes small increases in breathing or heart rate *[brisk walking, carrying light loads, manual washing clothes, mopping of floor, gardening at home]* for at least 10 minutes continuously?

A.	No 1
B.	Yes 2

24. In a typical week, on how many days do you do moderate-intensity activities as part of your work?

........................days

25. How much time do you spend doing moderate-intensity activities at work on a typical day?

...................hour: minutes

26. Do you walk or use a bicycle *(pedal cycle)* for at least 10 minutes continuously to get to and from places?

A.	No 1
B.	Yes 2

27. In a typical week, on how many days do you walk or bicycle for at least 10 minutes continuously to get to and from places?

........................days

28. How much time do you spend walking or bicycling for travel on a typical day?

………....................hour: minutes

29. Do you do any vigorous-intensity sports, fitness or recreational (leisure) activities that cause large increases in breathing or heart rate *[running or football]* for at least 10 minutes continuously?

A.	No 1
B.	Yes 2

30. In a typical week, on how many days do you do vigorous-intensity sports, fitness or recreational (leisure) activities?

.................days

31. How much time do you spend doing vigorous-intensity sports, fitness or recreational activities on a typical day?

.................hours

32 Do you do any moderate-intensity sports, fitness or recreational (leisure) activities that cause a small increase in breathing or heart rate *[brisk walking, cycling, swimming, volleyball, badminton, yoga]* for at least 10 minutes continuously?

A.	No 1
B.	Yes 2

33. In a typical week, on how many days do you do moderate-intensity sports, fitness or recreational (leisure) activities?

........................days

34. How much time do you spend doing moderate-intensity sports, fitness or recreational (leisure) activities on a typical day?

.....................hours

35. How much time do you usually spend sitting or reclining on a typical day?

......................hours

36. In an average, how long do you sleep in a day (24 hrs) **?**

A.	Less than 4 hrs..1
B.	4-6 hrs 2
C.	6-8 hrs 3
D.	8-10 hrs..4
E.	More than 10 hrs.......................................5

37. Do you did/do the shift work that involve night duty (like security work, driving, health work)**?**

A.	No 1
B.	Yes 2

38. Do you have any mental health problem or taking medicine related to that**?**

A.	No 1
B.	Yes 2

39. If yes, how long have you been suffering from mental problem **?**

A.	............years……..months………..days
B.	Do not know....................................77

Section B: Food frequency questionnaire (FFQ)

Please select one if you have taken following food items in the past one year											
**Food items**	**Unit**	**Size of the unit (S/M/L)**	**Never or < once a month**	**1-3/ month**	**Once a week**	**2-4/ week**	**5-6/ week**	**Once a day**	**2-3/ day**	**4-5 / day**	**6+/day**
**1. Drinking good items (5)**											
Milk/black tea with sugar			1	2	3	4	5	6	7	8	9
Milk/black coffee with sugar			1	2	3	4	5	6	7	8	9
Fruits juice			1	2	3	4	5	6	7	8	9
Soft drinks (Coco cola, Fanta, Sprite, etc)			1	2	3	4	5	6	7	8	9
Alcohol (Wine, vodka, bear, whisky)			1	2	3	4	5	6	7	8	9
Others			1	2	3	4	5	6	7	8	9
**2. Milk and milk products (5)**											
Milk (whole/skim/powder)			1	2	3	4	5	6	7	8	9
Yogurt			1	2	3	4	5	6	7	8	9
Butter milk/ Lassi			1	2	3	4	5	6	7	8	9
Paneer/Cheese			1	2	3	4	5	6	7	8	9
Milk sweets			1	2	3	4	5	6	7	8	9
Others..................			1	2	3	4	5	6	7	8	9
**How long have you been taking youghurt and milk?** A.......................year................... B. Don't know............................77											
**3. Cereals product (17)**											
White rice (Bhaat)			1	2	3	4	5	6	7	8	9
Beaten/puffed rice (Cheeura/Bhuja)			1	2	3	4	5	6	7	8	9
Corn porridge (Aato/Dhido)			1	2	3	4	5	6	7	8	9
Boiled/barbequed green Maize			1	2	3	4	5	6	7	8	9
Roasted Maize/flour from grinned roasted maize (Saatu)			1	2	3	4	5	6	7	8	9
Oats/Musheli			1	2	3	4	5	6	7	8	9
Millet bread/porridge (Kodo/Fapar ko roti/Dhido)			1	2	3	4	5	6	7	8	9
Wheat local bread/ Porridge (Gilo roti/Dhido)			1	2	3	4	5	6	7	8	9
Wheat bread (Tawa/sukkah Roti)			1	2	3	4	5	6	7	8	9
Dough bread/toast			1	2	3	4	5	6	7	8	9
Pasta/Chawmen/Noodle			1	2	3	4	5	6	7	8	9
Samosha (Singhada)			1	2	3	4	5	6	7	8	9
Puri/Paratha			1	2	3	4	5	6	7	8	9
Jeri/sweets			1	2	3	4	5	6	7	8	9
Dalmod/Namkeen			1	2	3	4	5	6	7	8	9
MoMo (chicken/buffalo)			1	2	3	4	5	6	7	8	9
MoMo (Vegetables)			1	2	3	4	5	6	7	8	9
Others			1	2	3	4	5	6	7	8	9
**For getting complete cerel consumption histry, please ask respondent that what they eat in breakfast and snaks during whole day besides lunch and dinner.**											

Food items	Unit	Size of the unit (S/M/L)	Never or < once a month	1-3/ month	Once a week	2-4/ week	5-6/ week	Once a day	2-3/ day	4-5 / day	6+/day
**4. Lentil/Pulses/beans (10)**											
**What consistency of cooked daal usually do you eat?** A. Semi solid (hardly pour from bowl)....................1<br>B. Medium (easily pour from bowl) ....2 C. liquid (like a water rice) 3											
Lentils (Masuro daal)			1	2	3	4	5	6	7	8	9
Yellow gram (Arhar Daal)			1	2	3	4	5	6	7	8	9
Black Gram (Maas Daal)			1	2	3	4	5	6	7	8	9
Bengal gram (Chana Daal)			1	2	3	4	5	6	7	8	9
Mixed pulses (Mixed Daal)			1	2	3	4	5	6	7	8	9
Peas (Kerau Daal)			1	2	3	4	5	6	7	8	9
Rajmah/Beans curry			1	2	3	4	5	6	7	8	9
Benghal gram (Channa boil)			1	2	3	4	5	6	7	8	9
Mixed beans/pulses (Quanti)			1	2	3	4	5	6	7	8	9
Roasted Soybean			1	2	3	4	5	6	7	8	9
Others ..			1	2	3	4	5	6	7	8	9
**5. Egg/Meat/Fish (6)**											
Boiled egg/ Umlet			1	2	3	4	5	6	7	8	9
Chicken meat- fried/gravy			1	2	3	4	5	6	7	8	9
Goat meat- fried/gravy			1	2	3	4	5	6	7	8	9
Buffalo meat- dried/fried/gravy			1	2	3	4	5	6	7	8	9
Pig meat- fried/gravy			1	2	3	4	5	6	7	8	9
Fish- dried/fried/gravy			1	2	3	4	5	6	7	8	9
Others			1	2	3	4	5	6	7	8	9
**6. Vegetables (5)**											
Green leafy vegetables curry (Mustard, Spinach, pumpkins, fenugreek leaf, …)			1	2	3	4	5	6	7	8	9
Green Beans vegetables curry (beans, long beans, green peas)			1	2	3	4	5	6	7	8	9
Other vegetables curry (Cauliflower, Cabbage, lady's finger, pumpkin, guard, tomatoes)			1	2	3	4	5	6	7	8	9
Potato cooked in curry			1	2	3	4	5	6	7	8	9
Salad (Cucumber, Onion, Radish , carrot)			1	2	3	4	5	6	7	8	9
**Do you cook daal and curry when you cooked meet in your family kitchen ? A. Yes B. No**											
**7. Spices and condiments (2)**											
Garlic and onion			1	2	3	4	5	6	7	8	9
Spices, chillis, turmeric			1	2	3	4	5	6	7	8	9
8. **Fruits (8)**											
Dry fruits (raisins and dates)											
Dry fruits (nuts and others)											
Banana			1	2	3	4	5	6	7	8	9

Food items	Unit	Size of the unit (S/M/L)	Never or < once a month	1-3/ month	Once a week	2-4/ week	5-6/ week	Once a day	2-3/ day	4-5 / day	6+/day
Apple			1	2	3	4	5	6	7	8	9
**Note: Please ask this question: During season, how often do you eat below mentioned fruits in a week or month?**											
Mango			1	2	3	4	5	6	7	8	9
Orange			1	2	3	4	5	6	7	8	9
Pomegranate			1	2	3	4	5	6	7	8	9
Other seasonal fruits (Grapes. Papaya, Guava, Pine apple, Watermelon, Pear, lychee and others)			1	2	3	4	5	6	7	8	9
**9. Others food (3)**			1	2	3	4	5	6	7	8	9
Pizza			1	2	3	4	5	6	7	8	9
Burgers			1	2	3	4	5	6	7	8	9
Any type of chips			1	2	3	4	5	6	7	8	9
**10. Fats/Oil (2)**											
Animal Ghee/fat			1	2	3	4	5	6	7	8	9
Vegetables butter/ghee			1	2	3	4	5	6	7	8	9
Others			1	2	3	4	5	6	7	8	9

Did you use to take any other food more than once a week ? A. yes B. No

S.N.	Food items	Size of food	Frequency per week

Section C: Fat and oil specific questionnire

1. Usually which type of following oil or animal fat/ghee do you use for cooking food in your family kitchen?

A.	Mustard oil 1
B.	Sunflower oil 2
C.	Soyabean oil 3
D.	Plant ghee/butter 4
E.	Animal (cow/buffalo) butter/ghee 5
च	Animal fat...6
छ	Others (please state)..................................88

2. How long have you been using above you mentioned oil/fat**?**

A.	.........................years............................1
B.	Don't know 77

3. Have you been changing above you mentiond cooking oil/fat time to time **?**

A.	No 1
B.	Yes 2
C.	Sometimes..3
D.	Don't know 77

4. If yes what kind of alternate oil do you use?

A.	Usually mustard oil and sometimes others...1
B.	Usually sunflower oil and sometimes others. 2
C.	Usually soyabean oil and sometimes others.. 3
D.	Others (please state)................................88

5. How long have you been changing above you mentioned alternative oil/fat**?**

A.	.........................years...........................1
B.	Don't know 77

6. Since past have you change the consumption of fat/oil?

A.	Increased 1
B.	Decreased 2
C.	No change 3
D.	Don't know 77

7. How long have you changed cooking oil/fat**?**

A.	.........................years............................1
B.	Don't know 77

8. If you buy 1 liter oil, for how long, in an average, do you cook food from this in your family's shared kitchen?

A.	.........................years............................1
B.	Don't know 77

(1 muthi = 50 ml, 1 quarter = 125 ml**,** 5 muthi = 250 ml, 1 maana = 500 ml**)**
..................ml

9. How many people do you eat in combine kitchen?

< 3 year.......................1	3-5 years......................2
5-7 years......................3	7-9 years......................4
9-12 years...................5	12-21 years..................6
>21 years.......................7	

10. Do you eat ghee with other food?

A.	No 1
B.	Yes 2
C.	Sometimes 3
D.	In past yes, now quieted........................4

11. If yes, which type of ghee do you prefer?

A.	Butter 1
B.	Ghee 2
C.	Plant ghee (Peanut Butter) 3
D.	Others (please state)..........................88

12. How long have you been eating above you mentioned ghee**?**

A.	.........................years............................1
B.	Don't know 77

13. When you buy meet, if you see animal fat, what you will do?

A.	Throw whatever seen 1
B.	Totally consume 2
C.	First boil and then cook……………......3

14. How often do you eat dried and fried meat (sukuti and sekuwa)?

A.	Never...1
B.	Less than once a week........................2
C.	1-3 times/week 3
D.	4-6 times/week 4
E.	Daily 5

15. How often do you consume deep fried food in your home ?<br>A. Never...1<br>B. Less than once a week.......................2<br>C. 1-3 times/week ....3<br>D. 4-6 times/week ....4<br>E. Daily 5	16. If yes, how long have you been consuming deep fried food?<br>A. ....years............................1<br>B. Don't know ....77<br>17. To what extent do you prefer fatty food, fried and deep fried food, yellow part of an egg?<br>Strongly lik…………..5 Like………………4<br>Neutral ……………...3 Dislike …………….2<br>Strongly dislike……...1

Name :.. Adress................................ Phone No:.......................................

Section D: Sociodemographic information

1. Cast/ethnicity**:**
   - A. Bramin/Chhetri/Thakuri 1
   - B. Newar 2
   - C. Janajati 3
   - D. Dalit 4
   - E. Tarai/Madesh Caste............................ 5
   - F. Others 88
2. Age**:** .... Years
3. Sex**:**
   - A. Female 1
   - B. Male 2
   - C. Others 3
4. Marital status**:**
   - A. Bachelor 1
   - B. Married 2
   - C. Divorced/sepereted 3
   - D. Widow/widowess 4
5. Occupation**:**
   - A. Household work ……………………… 1
   - B. Agricultural/labour work …………..... 2
   - C. Regular job/teaching 3
   - D. Self employed/ small-scale business 4
   - E. Largescale business (>15 staffs............ 5
   - F. Retired 6
   - G. Unemployed 7
6. Educational Level
   - A. Illiterate 1
   - B. Primary level 2
   - C. Lower secondary 3
   - D. Secondary 4
   - E. Higher secondary 5
   - F. College and above …........................... 6
7. Monthly family income
   - A. <10000 1
   - B. 10000-200000 2
   - C. 20000-30000 3
   - D. 30000-40000 4
   - E. 40000-50000 5
   - F. > 50000 6
   - G. Do not know 77

Section: E Anthropometric and other measurement

1	Height cm		8	Blood sugar	
2	Weight kg		9	Total cholesterol	
3	BMI		10	Triglycerides	
4	Waist cm		11	HDL level	
5	Hip cm		12	LDL level	
6	Waist and Hip ratio		13	Blood grouping	
7	Blood pressure	.......................mm of hg	14	CPK MB	
