# Supplementary material for: Dietary nutrients of relative importance associated with coronary artery disease: Public health implication from random forest analysis
Source: PLoS One. 2020 Dec 10;15(12):e0243063. doi: 10.1371/journal.pone.0243063 (PMC7728256; doi:10.1371/journal.pone.0243063)
Supplement: S4 File — (PDF) [file pone.0243063.s008.pdf]

Government of Nepal
Nepal Health Research Council (NHRC)
Estd. 1991

Ref. No.: 2995

10 June 2018

Mr. Til Bahadur Basnet
Principal Investigator
Nanjing Medical University

Ref: **Approval of thesis proposal** entitled **Relationship among dietary nutrients intake, cardiometabolic risk factors and coronary artery disease in Nepalese: A hospital based case control study**

Dear Mr. Basnet,

It is my pleasure to inform you that the above-mentioned proposal submitted on **1 June 2018** **(Reg. no. 308/2018)** please use this Reg. No. during further correspondence) has been approved by Nepal Health Research Council (NHRC) Ethical Review Board on **6 June 2018**.

As per NHRC rules and regulations, the investigator has to strictly follow the protocol stipulated in the proposal. Any change in objective(s), problem statement, research question or hypothesis, methodology, implementation procedure, data management and budget that may be necessary in course of the implementation of the research proposal can only be made so and implemented after prior approval from this council. Thus, it is compulsory to submit the detail of such changes intended or desired with justification prior to actual change in the protocol. Expiration date of this proposal is **August 2019.**

If the researcher requires transfer of the bio samples to other countries, the investigator should apply to the NHRC for the permission. The researchers will not be allowed to ship any raw/crude human biomaterial outside the country; only extracted and amplified samples can be taken to labs outside of Nepal for further study, as per the protocol submitted and approved by the NHRC. The remaining samples of the lab should be destroyed as per standard operating procedure, the process documented, and the NHRC informed.

Further, the researchers are directed to strictly abide by the National Ethical Guidelines published by NHRC during the implementation of their project proposal and **submit progress report in between and full or summary report upon completion.**

As per your thesis proposal, the total research amount is **Self-Funded** and accordingly the processing fee amounts to **NRs 10,000.** It is acknowledged that the above-mentioned processing fee has been received at NHRC.

If you have any questions, please contact the Ethical Review M & E Section at NHRC.

Thanking you,

Nirbhay Kumar Sharma
Acting Administrative Chief
