# Supplementary material for: Dietary nutrients of relative importance associated with coronary artery disease: Public health implication from random forest analysis
Source: PLoS One. 2020 Dec 10;15(12):e0243063. doi: 10.1371/journal.pone.0243063 (PMC7728256; doi:10.1371/journal.pone.0243063)
Supplement: S5 File — (PDF) [file pone.0243063.s009.pdf]

Reporting checklist for case-control study.

Based on the STROBE case-control guidelines.

Instructions to authors

Complete this checklist by entering the page numbers from your manuscript where readers will find each of the items listed below.

Your article may not currently address all the items on the checklist. Please modify your text to include the missing information. If you are certain that an item does not apply, please write "n/a" and provide a short explanation.

Upload your completed checklist as an extra file when you submit to a journal.

In your methods section, say that you used the STROBE case-controlreporting guidelines, and cite them as:

von Elm E, Altman DG, Egger M, Pocock SJ, Gotzsche PC, Vandenbroucke JP. The Strengthening the Reporting of Observational Studies in Epidemiology (STROBE) Statement: guidelines for reporting observational studies.

		Reporting Item	Page Number
**Title and abstract**			
Title	#1a	Indicate the study’s design with a commonly used term in the title or the abstract	1
Abstract	#1b	Provide in the abstract an informative and balanced summary of what was done and what was found	2
**Introduction**			
Background / rationale	#2	Explain the scientific background and rationale for the investigation being reported	3-4
Objectives	#3	State specific objectives, including any prespecified hypotheses	5
**Methods**			
Study design	#4	Present key elements of study design early in the paper	5

Setting	#5	Describe the setting, locations, and relevant dates, including periods of recruitment, exposure, follow-up, and data collection	5
Eligibility criteria	#6a	Give the eligibility criteria, and the sources and methods of case ascertainment and control selection. Give the rationale for the choice of cases and controls. For matched studies, give matching criteria and the number of controls per case	5-6
Eligibility criteria	#6b	For matched studies, give matching criteria and the number of controls per case	6
	#7	Clearly define all outcomes, exposures, predictors, potential confounders, and effect modifiers. Give diagnostic criteria, if applicable	6-9
Data sources / measurement	#8	For each variable of interest give sources of data and details of methods of assessment (measurement). Describe comparability of assessment methods if there is more than one group. Give information separately for cases and controls.	6-9
Bias	#9	Describe any efforts to address potential sources of bias	9
Study size	#10	Explain how the study size was arrived at	6 (model construction)
Quantitative variables	#11	Explain how quantitative variables were handled in the analyses. If applicable, describe which groupings were chosen, and why	6-9
Statistical methods	#12a	Describe all statistical methods, including those used to control for confounding	10
Statistical methods	#12b	Describe any methods used to examine subgroups and interactions	No sub group analysis
Statistical methods	#12c	Explain how missing data were addressed	'n/a'
Statistical methods	#12d	If applicable, explain how matching of cases and controls was addressed	6

Statistical methods	#12e	Describe any sensitivity analyses	'n/a'
**Results**			
Participants	#13a	Report numbers of individuals at each stage of study—eg numbers potentially eligible, examined for eligibility, confirmed eligible, included in the study, completing follow-up, and analysed. Give information separately for cases and controls.	All participated
Participants	#13b	Give reasons for non-participation at each stage	'n/a'
Participants	#13c	Consider use of a flow diagram	6
Descriptive data	#14a	Give characteristics of study participants (eg demographic, clinical, social) and information on exposures and potential confounders. Give information separately for cases and controls	11
Descriptive data	#14b	Indicate number of participants with missing data for each variable of interest	‘n.a’
Outcome data	#15	Report numbers in each exposure category, or summary measures of exposure. Give information separately for cases and controls	11-13
Main results	#16a	Give unadjusted estimates and, if applicable, confounder-adjusted estimates and their precision (eg, 95% confidence interval). Make clear which confounders were adjusted for and why they were included	14
Main results	#16b	Report category boundaries when continuous variables were categorized	11
Main results	#16c	If relevant, consider translating estimates of relative risk into absolute risk for a meaningful time period	Not relevant
Other analyses	#17	Report other analyses done—e.g., analyses of subgroups and interactions, and sensitivity analyses	'n/a'
**Discussion**			
Key results	#18	Summarise key results with reference to study objectives	16-17

Limitations	#19	Discuss limitations of the study, taking into account sources of potential bias or imprecision. Discuss both direction and magnitude of any potential bias.	20
Interpretation	#20	Give a cautious overall interpretation considering objectives, limitations, multiplicity of analyses, results from similar studies, and other relevant evidence.	20
Generalisability	#21	Discuss the generalisability (external validity) of the study results	20
**Other Information**			
Funding	#22	Give the source of funding and the role of the funders for the present study and, if applicable, for the original study on which the present article is based	'n/a'

Notes:

- 12b: No sub group analysis The STROBE checklist is distributed under the terms of the Creative Commons Attribution License CC-BY. This checklist was completed on 24. November 2019 using https://www.goodreports.org/, a tool made by the EQUATOR Network in collaboration with Penelope.ai
