# Supplementary material for: Dietary nutrients of relative importance associated with coronary artery disease: Public health implication from random forest analysis
Source: PLoS One. 2020 Dec 10;15(12):e0243063. doi: 10.1371/journal.pone.0243063 (PMC7728256; doi:10.1371/journal.pone.0243063)
Supplement: Attachment — Submitted filename: Review. PONE-D-20-07225.docx [file pone.0243063.s012.docx]

**Review**

**Manuscript Number:** PONE-D-20-07225
**Manuscript Title:** Dietary nutrients of relative importance associated with coronary artery disease: Public health implication from random forest analysis

**Summary of study**

This is a retrospective case-control study examining the difference in calorie consumption and intake of 18 nutrients between those with established coronary artery disease (CAD) (*n*=306) versus a control (*n*=306). The purpose of the study is to establish associations between the consumption of specific nutrients and CAD in the Nepalese population; associations that the authors believe ought to inform future RCTs/prospective cohort studies and future health policy.

It examined a total of 612 patients from the Shahid Gangalal National Heart Centre, Nepal with case-control matched via age and gender. Patients were recruited to the study following admission to the hospital due to a suspected coronary problem (recent suspected myocardial infarction, an exercise induced positive stress test result, or who elected to have an angiography). Patients were assigned to the ‘case’ group if they showed evidence of severe stenosis (>70%). Control subjects were patients who upon arrival were found to have ‘normal’ angiographic results (authors should specify exactly what this means – the flow chart in Fig 1 gives the impression of 0% stenosis) or who were found to have no ECG abnormalities following an exercise stress test. According to the authors, those with intermediate stenosis (<70% obstruction) were excluded from the study, presumably any patient with evidence of stenosis ranging from 1–69%.

Data on nutrient consumption over the previous 12-months for each participant was derived from an EPIC food frequency questionnaire. Authors calculated average daily calorie consumption and selected 18 nutrients for analysis – total fat, total carbohydrate, total protein, fibre, calcium, phosphorous, iron, zinc, thiamine, riboflavin, niacin, Vitamin C, β-Carotene, Vitamin A, polyunsaturated fatty acids (PUFA), monounsaturated fatty acids (MUFA), saturated fatty acids (SFA), and cholesterol. Data on known risk factors (diabetes; dyslipidaemia; hypertension; obesity based on BMI; obesity based on central obesity via waist–hip ratio), and data on behaviours believed to be associated with CAD (alcohol consumption; smoking status; and physical activity level) were also recorded for each patient.

Using conditional multivariable logistic regression, authors report two statistically significant positive associations after controlling for known risk-factors (model-2): (i) a positive relationship with total fat intake (OR 1.13, 95% CI 1.05–1.21, *P*≤0.001); (ii) a positive relationship with dietary cholesterol intake (OR 1.06, 95% CI 1–1.12, *P*=0.02) – note, however, the inclusion of 1 in the confidence interval. **The study also found statistically significant differences (inverse associations) between those with CAD and those without in terms of intakes of: (i) total carbohydrate intake (OR 0.93, 95% CI 0.86–0.99, *P*=0.04); (ii) calcium intake (OR 0.96, 95% CI 0.94–0.99, *P*=0.003); (iii) zinc intake (OR 0.88, 95% CI 0.79–0.98, *P*=0.02); (iv) niacin intake (OR 0.8, 95% CI 0.7–0.91, *P*≤0.001); (v) β-Carotene intake (OR 0.93; 95% CI 0.9–0.97, *P*=0.001).**

**Following this, the authors use a random forest regression to adjust for collinearity between the variables, which the authors believe allow them to discern the “five topmost important nutrients…linked with CAD: β-Carotene, fat, cholesterol, vitamin C, and fibre intakes”. However, the authors need to provide substantially more detail in the description of results and methodology used for this than is reported here. As it is, I’m not entirely sure what to make of these results.**

**They then proceed to offer a superficial comparison of their findings with the existing literature. This requires extensive revision, however, and the authors need to ensure they avoid committing citation bias here in regards to some of their claims. To do this, they need to show a greater understanding of the conflicting results of large RCTs, prospective cohort studies, and recent meta-analyses. Fortunately, for each of the nutrients examined here, there is an extensive literature on their relationship with CAD/CVD – so the authors really need to engage with this literature.**

**The authors conclude by claiming: “We conclude that dietary β-Carotene, total fat and oil, cholesterol, vitamin C, and fiber** **in the Nepalese population”.**

**However, I believe, the following points must be addressed before this study can be published:**

**Revisions and problems**

1. **Ethics**

Before publication, the authors need to include more details about ethical approval. PLOS ONE’s policy on this is as follows:

Human Subject Research (involving human participants and/or tissue)

- Give the name of the institutional review board or ethics committee that approved the study
- Include the approval number and/or a statement indicating approval of this research
- Indicate the form of consent obtained (written/oral) or the reason that consent was not obtained (e.g. the data were analysed anonymously))

The authors state that this study was approved by the Nepal Health Research Council (NHRC). Is the value given here (308/2017-18) the study registration code? Is this associated with certification of ethical clearance by the NHRC ethics committee? Could the authors please attach the appropriate ethical approval documentation as a supplementary file; a letter from the NHRC stating this clearance was provided will suffice. As the authors state that they have received written informed consent by every study participant, could the authors also go into some detail about whether those participants were informed their data (anonymised) would be available open access?

2. **Data availability**

The authors must supply *all* data associated with their analyses. The authors have claimed all relevant data are supplied in the paper or supplementary files, but this is inaccurate. The results of this paper depend on the analysis of distribution data, and this is necessary for replication. PLOS ONE’s policy states – “PLOS journals require authors to make all data necessary to replicate their study’s findings publicly available without restriction at the time of publication”. Accordingly, the relevant data for every study participant on which this papers analysis depends must either be included as supplementary files or stored in an online data repository after patient data has been appropriately anonymised. These data could be provided in spreadsheets – for the 612 patients, this involves providing all data on control/case group membership, age, weight, gender, dyslipidaemia, hypertension, obesity, smoking, alcohol consumption, physical activity, calorie consumption, and all data regarding intake of the 18 examined nutrients. Indeed, in light of the following problems, it is the data collected by the authors here is probably the most important aspect of the study.

3. **Problems concerning data analysis**

**Multiple comparisons**

One major problem is accounting for multiple comparisons in this study. For example, Table 2 lists 19 variables (18 nutrients and total calorie consumption) each of which has been compared with a Wilcoxon rank-sum test – so the quoted significance levels at the very least need to be adjusted for the fact that 19 comparisons have been made. The same issue recurs throughout the analysis. The authors should seek to resolve this issue via appropriate statistical methods. I would also recommend that this study is referred to a statistical editor upon these revisions to ensure that this issue has been appropriately resolved.

**Multicollinearity**

Apart from the problem of multiple comparisons here there is also the problem that the data are not fully independent (multicollinearity) – specific micronutrients tend to be associated with other particular nutrients in different food types. Accordingly, it is important not to over interpret associations unless these issues are rigorously excluded. Accordingly, the authors should reflect more on this issue and adjust their methods/interpretations in light of this. One option here would be to extend their discussion of their random forest regression. Indeed, the paper would benefit from extending and deepening the description and results of this analysis, providing results on correlations between nutrient intakes and variance inflation factor. Again, I believe a statistical editor should be consulted.

**Other data analysis issues:**

In Table 1, the authors claim that the median age of authors is statistically significantly different between the case and control groups. The authors report the median age, interquartile range, and *P*-value as:

Case: 58 (50–65) | Control: 58 (50–65) | *P*=0.001

Not only is the median age the same, but so too are the interquartile ranges. Yet, despite this, the age difference is apparently statistically significant? The authors seem to interpret this result as meaningful:

“Because the age was matched in five year intervals in the study, the median age was 58 years, which was the same in both case and control groups, respectively, and still showing strong association (*P*=0.001).”

The authors claim that age was matched in 5-year intervals, but are we then to interpret these results as suggesting that there is actually a significant difference in age between case-control matched pairs? As CAD is strongly associated with age in previous research, this is important to clarify. To do this, the authors need to report the age distribution data for both groups as a supplementary file.

For the analysis in Table 2, the authors claim “as most of the study data were not normally distributed, median and interquartile range of nutrients are presented”. The authors should, therefore, include in their supplementary material the actual data underpinning their analysis. At the very least, they must include the mean, range, and SD for each nutrient, so the reader can understand exactly what the distribution of these data actually are. Indeed, the data provided in the paper is insufficient to replicate the necessary results reported, despite the authors declaration.

**Statistical rhetoric**

The authors highlight a “highly significant” finding (p.9), this language is inappropriate and should be replaced. A result is simply *either* significant or non-significant and this is determined by whatever threshold of significance the authors deem necessary.

4. **Problems of variable selection and measurement**

**Nutrient selection**

The authors select 18 nutrients to examine here, but why these specific nutrients are analysed is not adequately justified. Accordingly, the authors should make clearer why these items were selected for analysis.

In the supplementary file, a list of common foods consumed is provided. This raises further questions about why the authors chose only to analyse the variables they selected in this paper because other variables appear possible to derive from their data. For example, I see no reason why the amount of sugars in the diet couldn’t be calculated from the listed food items, so why isn’t this examined in the paper? Similarly, their decision to use total carbohydrate intake as a variable without breaking this down into refined and complex carbohydrates appears strange and problematic, particular because the authors acknowledge in the paper that there are important differences between these. Why then didn’t the authors calculate these?

In its current state, this Table of food items is both uninformative and misleading. It also raises further questions about how nutrient intakes were measured. Patients were asked about milk consumption, but the milk category does not clarify whether respondents were asked specifically about the amount of full, semi, or skimmed milk consumed, which would be necessary to understand fat content and fatty acid profiles, or whether this was a single category. Further questions about how the quantities of PUFA, MUFA, and SFA were calculated arise in regards to several of the vague categories, such as “vegetable oils”. Accordingly, the authors should include the specific dietary survey actually provided to patients. Furthermore, supplying the average amount of each food consumed by cases and controls for each item would shed more light on dietary habits. As recent research suggests different whole foods might have different effects on lipid profiles and thereby atherosclerosis, these data are important to report. At the very least the authors need to make available the intakes of each nutrient examined in this study per patient.

Other questions that arise are why were PUFA here considered as a single group and not split further into Omega-3 and Omega-6 variants? Why was the intake of trans-fats not measured? Thus, the authors need to revise the manuscript to give the reader a clearer understanding of the theoretical justification for the selection of the variables. As there is a voluminous literature on the relationship between diet and atherosclerosis/CHD/CVD extending back to the early 20^th^ century, there is a wonderfully rich literature to draw from.

**Self-reported nutritional data**

As all the nutritional data are all self-reported, the authors should include a clearly discussion in regards to their reliability given the known problems with this kind of data. I suspect there is a problem here. From Table 2, it appears that total daily nutritional intake was virtually the same in the two groups – despite the significantly ***higher*** incidence of obesity in the control group.

5. **Referencing**

In-text references in this paper appear occasionally only loosely related to the claims they are purported to be associated with. For example, reference number 2 is inserted after the following sentence:

“In Nepal, 30% of total death was related to cardiovascular disease (2).”

Yet, reference 2 is a paper by Rankinen *et al.* (2015), and nowhere in this paper is this claim made. Another reference chosen at random, reference 20, is used to support the authors claim that:

“Besides cholesterol is also an independent risk factor of CAD according to the lipid theory”

The paper referenced nowhere discussed ***dietary*** cholesterol. It is a paper examining, as the title suggests, the “Relationships Between Components of Blood Pressure and Cardiovascular Events in Patients with Stable Coronary Artery Disease and Hypertension”. The only mention of cholesterol in this paper is HDL-C and LDL-C – that is, cholesterol bound in particular classes of lipoproteins carried in the blood.

If the authors make the rest of the revisions outlined, I will examine each of the references of this paper. So my recommendation would be to go through each reference and ensure it is relevant to the claim being made. As discussed, the authors also need to ensure they have adequately represented the state of research in relation to their claims. If the article is resubmitted, I’ll check each.

6. **Flawed study design**

However, there is one problem that may undermine the point of revising this manuscript. The authors have a case-matched control group – but the control group are not healthy individuals, but patients with other health conditions and cardiovascular symptoms. This is clearly evident by the way the authors chose to enrol patients – all patients were being examined because of suspected coronary problems. This makes it impossible to talk of differences between these groups in terms of risk factors.

For example, looking at Table 1, the ‘control’ group has a significantly higher incidence of obesity and central obesity – but it would be obviously wrong to conclude that obesity and central obesity are protective against cardiovascular disease. Here we’re seeing a stratification of phenotypic characteristics between two different patient groups, and from this we can’t conclude *anything* at all about risk.

This might also explain the extremely strange finding that the number of hypertensives was roughly the same in both the control and case groups - Control: 143 hypertensives (~46.7%) | Case: 142 hypertensives (~46.4%). As hypertension is one of the key known risk factors in the development of CAD/CVD and extensively supported in the literature, this finding requires a lot more reflection. Why were hypertensives so common in the control group? This control group had apparently no evidence of stenosis – so this seems to be quite an important avenue to explore what went on here.

Accordingly, this design is inappropriate for the authors stated intention: “The present case-control study was designed to determine the association of dietary nutrients with CAD in the Nepalese population”.

If this study is to be published, the authors need to somehow explain why this control group can be considered representative of a broader population. Later in the paper the authors do highlight the results may have been biased due to “the selection of the control group from the outdoor patients from the same hospital where more hypertensive patients come for their heart check-up”, but this seems to *critically* undermine the entire results of this study.
