# Supplementary material for: Dietary nutrients of relative importance associated with coronary artery disease: Public health implication from random forest analysis
Source: PLoS One. 2020 Dec 10;15(12):e0243063. doi: 10.1371/journal.pone.0243063 (PMC7728256; doi:10.1371/journal.pone.0243063)
Supplement: Attachment — Submitted filename: ResponseLetter_PloS One.docx [file pone.0243063.s013.docx]

**Response Letter**

July 30, 2020

To,

Dear Academic editors and Reviewers,

We highly appreciate comments of academic editor and both the reviewers in our manuscript entitled " ***Dietary nutrients of relative importance associated with coronary artery disease: Public health implication from random forest analysis***" (PONE-D-20-07225). We have modified the manuscript; accordingly, there has been a visible improvement in our manuscript after incorporating the comments. The responses to the comments are listed below.

We hope this manuscript will be acceptable for publication in your reputed journal.

Sincerely,

Til Bahadur Basnet

Ph.D. Candidate (Epidemiology and health statistics)

………………………………………..

1. **Academic editor comments**

- The manuscript lacks important methodological details needed for correct interpretation of the study. Please describe in better depth: (i) why your preferred to use “Random forest analysis”, and why both conditional LR and Random Forest Analysis were employed together to answer the same research question? (ii) Why these specific dietary nutrients were selected for analysis (why not others)? (iii) How did you select potential confounders for adjustment? (iv) How was the sample size of 306 cases and 306 controls determined? (v) what was the justification for developing two different multivariable models (model 1 and model 2) to explain an outcome while it is possible to have a single model with a better fit?

**Response:** Thank you so much for all the comments, which were very valuable and encouraging. We believe that we have improved the methodological details for correct interpretation of the study addressing the quarries raised.

(i) why your preferred to use “Random forest analysis”, and why both conditional LR and Random Forest Analysis were employed together to answer the same research question?

Random forest (RF) analysis is an algorithm based technique that not only picks up the important variables but also useful technique when the data have normality as well as multicollinearity problem. RF is a useful technique when data have relatively fewer observations because the analysis is based on an ensemble of classification trees in which it split the data into several nodes that maximized the homogeneity in each group, the random forest assembled hundreds more classification trees with a selection of correlates randomly. However, it cannot determine the strength of association. In contrast, conditional LR is the correct method to determine the strength of association in matched case control study. Therefore, we preferred both analyses in our study.

(ii) Why these specific dietary nutrients were selected for analysis (why not others)?

Upon review of the literature, dietary nutrients that we selected were associated with coronary artery disease (CAD) with the discrepancy in results in different populations across the globe. And we calculated nutrient intake based on "Nepal food composition table 2017," which lacks other specific nutrients like biotin, magnesium, selenium, and others related to CAD.

(iii) How did you select potential confounders for adjustment?

Although there are several newer techniques like a-priori change (e.g., 10% or 15%) in the effect estimate criteria, bias-variance tradeoff, directed acyclic graph (DAG), we adopted conventional way of a p-value of cut off in the statistical model. After bivariate analysis (Wilcoxon sign-rank sum test for continuous but non-parametric paired data and McNemar test for dichotomous paired data analysis in matched case-control study ) [1], those variables which showed *p-*value less than 0.1 ( usually ranges from 0.05 up to 0.2) in bivariate analysis has been considered as potential confounders for adjustments.

(iv) How was the sample size of 306 cases and 306 controls determined?

The sample size was calculated for matched case-control study with group of unequal sample size taking Z_α_=0.05, Z_β_=0.8, prevalence of exposure = 0.19 (prevalence of smoking in the Nepalese population [2]), minimum detectable OR = 1.75. Using the formula developed by Schlesselman, the minimum sample size was 279. After adding a 10% non-response rate, the sample size was 306 in each group.

However, the present work was to construct a model using conditional logistic regression analysis. For multivariable analysis, the number of observations, in general, is ten times the number of predictors was suggested [3]. In this study, 19 nutrients variables and ten socio-demographic and cardio-metabolic risk factors were considered. Indeed, we selected only 23 predictors in the initial step of model construction. In the final model, only 12 variables were considered significantly associated with the outcome variables. Thus, we believe our sample size was considered enough for the present research work.

(v) What was the justification for developing two different multivariable models (model 1 and model 2) to explain an outcome while it is possible to have a single model with a better fit?

Thank you for your suggestion to develop a single model with a better fit. We have constructed a single energy-adjusted parsimonious multivariate model with a better fit by stepwise backward deletion process. And the results from the final model were presented in table 4.

- It is not clear how some of the variables (e.g. physical exercise, smoking, alcohol use) were measured and categories. Can you please add a sub-section (under the methods section) that describes the variables of the study along with the measurement and classifications employed?

We acknowledge your suggestions. So, we included the operational definition of conventional variables (e.g., physical exercise, smoking, alcohol use) as a sub-section under the methods section and described how they were measured and categorized.

- Can you explain further how you detected very significant statistical difference in the age of the cases and controls after having exactly the same median and IQR values?

Our study was matched case-control study, and we exactly matched the “sex," but we matched the “age” at an interval of 5 years. Because of interval matching and Wilcoxon-sign ranked test for matched paired non-parametric data, “age” showed a significant association with outcome.

- It seems the authors have already published on risk factors of CHD using the same dataset before (https://pubmed.ncbi.nlm.nih.gov/31588344/). Please describe in the background section how this paper is different from the earlier and why it was not possible to report the findings of the two papers together.

The paper which we published was the “association of smoking with CAD." If we included the dietary nutrients in that article, that would be lager in word count, which was beyond an essential requirement for the journal. Also, the paper would not be specific and exciting to the reader. Therefore, because of the words limit in the journal and specific detailed description of factors associated, it was not possible to include the dietary factors in the previous manuscript.

- Most of the reported ORs only have borderline statistical significance. Based on these findings, would that be possible to given strong practical recommendations? Can you discuss the issue further?

Unlike conventional risk factors (such as hypertension, diabetes, smoking), there was still a discrepancy in findings of the association of dietary nutrients with CAD in different populations across the world. Furthermore, several studies have reported relatively a higher effect size for conventional risk factors and smaller effect size for nutritional risk factors. We also observed some essential nutrients (beta-carotene, riboflavin, fiber, and vitamin C) that have not met the minimum recommended daily requirements (RDA) both in cases and controls. To confirm the effect of nutrients specific such as antioxidant vitamins on CAD, RCT and large cohort studies are required in the Nepalese population.

- As noted by both of the reviewers, please discuss the limitations of study in a better depth.

Thank you much for scrupulous perusing and valuable comments for making quality paper publishable in PLoS One, a world-famous journal. We included all the feedback provided by both reviewers, including the limitations of the study noted by reviewers.

**Other section-by-section comments
Abstract**

Report separately the sample size for cases and controls. It would also be good to provide the concise enrolment criteria for cases and controls.

Now, we included the sample size for cases and control in the abstract. Also, we have written the concise enrolment criteria for cases and controls.

**Background**

- This section is somehow superficial. Can you please add a paragraph that summarizes what is known about the dietary risk factors of CAD?

As per your suggestion, we have included one paragraph briefly describing the dietary risk factors of CAD.

**Methods and materials**

- Line 78-80: what about the 24-hr recall?
- Line 89-90: can you please describe the "24-h recall diet survey" in a better depth? In the results section, I don’t see any data on the results of this Criterion Validity assessment.
- Line 112: why did you use Wilcoxon sign ranked test for continuous variables? Was the distribution not normally distributed? Have you tried transformation before resorting to this non-parametric test?

We appreciate the comments that assisted a lot in improving our manuscript.

- 24-hr recall has been described in the manuscript.
- Before collecting the information from 306 cases and 306 controls, we carried out a survey among 115 OPD patients in the hospital to determine the validity and reproducibility of FFQ customized to Nepalese food items. Now the result has been incorporated as a supplementary file.
- Because the data were not normally distributed, we determined to apply a non-parametric test. We believe an interpretation of transformed data misleads our result in bivariate analysis. Wilcoxon sign-rank sum test for continuous but non-parametric paired data and McNemar test for dichotomous paired data are the correct tests for the analyses in matched case-control study [1].

**Results**

- Line 129-31: “This might be because of the selection of the control groups from the outdoor patients of the same hospital where more hypertensive patients come for their heart checkup”. This should rather be presented in the “discussion” section
- Table 1: “Hard” > “vigorous”
- Table 1: drinking alcohol and smoking: have you tried consider dose of exposure while defining these variables?
- Line 134-143: is this from the 24-hr recall or the food frequency questionnaire?
- Table 2: please clearly denote significant differences with “*”
- Line 145-48: it is good that you have used conditional logistic regression. But what was the variable/s conditioned in the analysis. if “age” was the conditioning variable, then why was it adjusted in the model?

Line 129-31: We appreciate the comments and have written it in the discussion section.

- We have changed "hard" into "vigorous."
- Our questionnaire was designed to collect the dose of exposure to alcohol use and smoking. However, because of inconsistency in responses, we resorted to categorical variables.
- The information mentioned in the Line 134-143 was from the food frequency questionnaire. We now described the source of information in the text.
- In Table 2, we now clearly denoted significant differences with “*”.
- Line 145-48: Matching in a case-control study does not control for confounding by the matching factors. A matched design may require controlling for the matching factors in the analysis [4]. In our study, we exactly matched the "sex," but we matched the "age" at an interval of 5 years. Because of interval matching and Wilcoxon-sign ranked test for paired data, “age" showed a significant association with outcome. Therefore, even though "sex" and "age" were conditioning variables in the study, we adjusted “age” in the model. However, it had been removed in step-wise backward deletion process while developing the single parsimonious model as per your suggestion.

**Journal Requirements:**

When submitting your revision, we need you to address these additional requirements.

1. Please ensure that your manuscript meets PLOS ONE's style requirements, including those for file naming. The PLOS ONE style templates can be found at

https://journals.plos.org/plosone/s/file?id=wjVg/PLOSOne_formatting_sample_main_body.pdf and

https://journals.plos.org/plosone/s/file?id=ba62/PLOSOne_formatting_sample_title_authors_affiliations.pdf

 We have prepared the manuscript according to PLOS ONE's style requirements, including those for file naming.

2. Please include additional information regarding the survey or questionnaire used in the study and ensure that you have provided sufficient details that others could replicate the analyses. For instance, if you developed a questionnaire as part of this study and it is not under a copyright more restrictive than CC-BY, please include a copy, in both the original language and English, as Supporting Information.

We are submitting the survey questionnaire in both the original language and English, as Supporting information.

3. Please amend the manuscript submission data (via Edit Submission) to include authors Ali Asghar Mirjat, Falak Zeb and Wiwik Indayati.

Now, we amended the manuscript submission data.

4. Your ethics statement must appear in the Methods section of your manuscript. If your ethics statement is written in any section besides the Methods, please move it to the Methods section and delete it from any other section. Please also ensure that your ethics statement is included in your manuscript, as the ethics section of your online submission will not be published alongside your manuscript.

We appreciated the advice to include the ethical statement. We have written it in the Method section of our manuscript.

5. Please include captions for your Supporting Information files at the end of your manuscript, and update any in-text citations to match accordingly. Please see our Supporting Information guidelines for more information: [http://journals.plos.org/plosone/s/supporting-information](about:blank).

We included the captions for supporting information files at the end of the manuscript and updated in-text citations.

**Reviewer #1:**

1) Innovation perspective the research is not very significant for the researchers globally. Most of the innovations are already available in the literature. As far my knowledge many studies already published these kinds of results. For example, Mahalle et. al. (2016): Association of dietary factors with severity of coronary artery disease. Authors in the manuscript (line no. 257) state “Finally, we found a consistent result with other published studies.”

Thank you so much for the comments. This matched case-control study was carried out to determine the association of dietary nutrients with CAD among Nepalese was the first case-control study. We believe that differences in the dietary habit of the different populations across space and time warrant a broader understanding of the dietary nutrients related to a specific disease.

2) The primary outcome of any case-control study is mainly based on the quality data. If the data is not good quality data then the observations on these data don’t confirm any firm statement. Based on interview questionnaire and the response of the person don’t strongly establish the dietary quantity and quality. If the diet would be provided by any good supply source who would maintain the quantity and quality of the food consumed by the patients, then, the data quality would be much better.

We acknowledge that the quality data are the most for the interpretation of the case-control study. Although a 24h dietary recall survey is considered a standard dietary survey tool, it can only collect the prier 24h diet information. Therefore, we used customized validated FFQ to collect the diet information during the past 12 months, and the average was calculated. Thus, we believe short term supply of quality food in the hospital or home would not have changed our entire data.

3) There is no external validation of the results. It majorly focused on Nepalese dataset but the research title is generic. External validation (dataset collecting from near countries such as India, Bhutan, Bangladesh) is required for the title otherwise title need to be changed to focus on Nepalese population perspective.

We appreciate for your suggestion. We have mentioned the study population in the methods section and external validation in the limitation of the study section.

4) The cohort is has been taken from one hospital and therefore, the results may be biased until the data has been taken in standard published process. At least 2 cohorts’ data is required for this study as it is based on face to face interview questions.

In Nepal, there are only two public hospitals, namely Shahid Gangalal National Heart Center and Monmon Cardiothoracic Treatment Center, which are specially equipped to treat heart-related health problems. We took permission from both hospitals and got ethical approval from the national health research council in Nepal. However, we reported very few cases (~5 cases in a month) from Monmon Cardiothoracic Treatment Center. We determined to carry out in Shahid Gangalal National Heart Center, a national level cardiac referral center in the country.

5) When we apply model’s then its strength needs to be checked by providing some metrics, such as, model’s stability by providing ROC curve, sensitivity, specificity, accuracy, f-score etc. These are clearly missing here.

Thank you for your suggestion. We have included the ROC curve and a comparison table about sensitivity, specificity, accuracy, and f-score of the two regression analysis models.

6) There are several standard algorithms compared to Random Forest (RF) regression are available. This study chose RF without giving proper justification.

We used random forest analysis because our data had a multicollinearity problem. Also, RF analysis is famous in predicting diseases in the medical field [5-7]. In the revised manuscript, we have provided the proper justification in statistical analyses of the "method" section.

7) Case: control is used 1:1 which is pretty rare in real scenarios. This study has lacks in producing right case and control samples. For example, it doesn’t tell how many patients have participated in the study and how some of them have been excluded from the study with proper logic. By using only Angiography (<70% stenosis) is not enough.

Although we have not reported the number of a non-participant in the study, we had collected the dietary information according to our predetermined inclusion and exclusion criteria that we mentioned in the "method" section.

8) In the discussion, some of the statements are contradict with the previous studies. But most of the cases, their rationalities are not explained clearly.

We believe that the discussion section has modified to contrast and support our study findings based on available literature.

9) Discussion section is not well organized to understand the study as stated in the title.

Our title is the relative importance of dietary nutrients related to CAD. We believe that we have discussed all nutrients associated with CAD in the study.

10) No justification has been given on selecting confounding variables for adjustment.

Although there are several newer techniques like a-priori change (e.g., 10% or 15%) in the effect estimate criteria, bias-variance tradeoff, directed acyclic graph, etc., we adopted conventional way of a p-value of cut off in the statistical model. After bivariate analysis (Wilcoxon sign-rank sum test for continuous but non-parametric paired data and McNemar test for dichotomous paired data analysis in matched case-control study ) [1], those variables which showed *p-*value less than 0.1 ( usually ranges from 0.05 up to 0.2) in bivariate analysis has been considered as potential confounders for adjustments.

11) For selecting confounding variables from one result to the next, there are several techniques like forward elimination, backward elimination could be used.

We used step-wise backward elimination technique while selecting confounding variables from one result to next.

12) There are many statements stated without giving proper references, e.g., line no. 27, 29, 44, etc. and so on.

We added the citation in the statements (line no. 27, 29, 44,) and also where references were missing.

13) The full questionnaire and data are not exposed to assess the results.

We have now submitted the full questionnaire and data as a supplementary file.

14) Source code of the models and data are available to reproduce the results.

We now have submitted the source code of R of the model and data as a supplementary file.

15) Some silly inconsistencies, like, line 144 states p<0.1.

While considering potential confounding factors, we have read that some epidemiological papers were taking variables not only p-value up to <0.2 but also the significant cofounding variables in other literature in the model development process. However, we now determined to resort to 0.05 as per your suggestion.

#Reviewer 2

1. Ethics
   Before publication, the authors need to include more details about ethical approval. PLOS ONE’s policy on this is as follows:

   "Human Subject Research (involving human participants and/or tissue)
   - Give the name of the institutional review board or ethics committee that approved the study
   - Include the approval number and/or a statement indicating approval of this research
   - Indicate the form of consent obtained (written/oral) or the reason that consent was not obtained (e.g. the data were analysed anonymously))"

   The authors state that this study was approved by the Nepal Health Research Council (NHRC). Is the value given here (308/2017-18) the study registration code? Is this associated with certification of ethical clearance by the NHRC ethics committee? Could the authors please attach the appropriate ethical approval documentation as a supplementary file; a letter from the NHRC stating this clearance was provided will suffice. As the authors state that they have received written informed consent by every study participant, could the authors also go into some detail about whether those participants were informed their data (anonymised) would be available open access?

Yes, the value given (308/2017-18) is the study registration code. Of course, this is associated with the certification of ethical clearance by the Nepal Health Research Council (NHRC) ethics committee. According to your suggestion, we are pleased to attach the ethical approval documentation as a supplementary file: a letter from the NHRC stating this clearance. However, we did not inform participants into some detail about their data (anonymised) would be available open access.

2. Data availability
The authors must supply all data associated with their analyses. The authors have claimed all relevant data are supplied in the paper or supplementary files, but this is inaccurate. The results of this paper depend on the analysis of distribution data, and this is necessary for replication. PLOS ONE’s policy states – “PLOS journals require authors to make all data necessary to replicate their study’s findings publicly available without restriction at the time of publication”. Accordingly, the relevant data for every study participant on which this papers analysis depends must either be included as supplementary files or stored in an online data repository after patient data has been appropriately anonymised. These data could be provided in spreadsheets – for the 612 patients, this involves providing all data on control/case group membership, age, weight, gender, dyslipidaemia, hypertension, obesity, smoking, alcohol consumption, physical activity, calorie consumption, and all data regarding intake of the 18 examined nutrients. Indeed, in light of the following problems, it is the data collected by the authors here is probably the most important aspect of the study.

Thank you for your suggestion. We have now submitted all the data in spreadsheets on control/case group membership, including all variables under study.

3. Problems concerning data analysis
Multiple comparisons:
One major problem is accounting for multiple comparisons in this study. For example, Table 2 lists 19 variables (18 nutrients and total calorie consumption) each of which has been compared with a Wilcoxon rank-sum test – so the quoted significance levels at the very least need to be adjusted for the fact that 19 comparisons have been made. The same issue recurs throughout the analysis. The authors should seek to resolve this issue via appropriate statistical methods. I would also recommend that this study is referred to a statistical editor upon these revisions to ensure that this issue has been appropriately resolved.

Our study was about variable selection and model construction. It was not about evaluating the association of each variable and the outcome, and nineteen nutrients were not the categories of a single variable. Each variable was separately tested with a Wilcoxon sign rank test for the selection of potential variables for model construction. Thus, we believe that there were not multiple comparison problems in our data set.

Multicollinearity:
Apart from the problem of multiple comparisons here there is also the problem that the data are not fully independent (multicollinearity) – specific micronutrients tend to be associated with other particular nutrients in different food types. Accordingly, it is important not to over interpret associations unless these issues are rigorously excluded. Accordingly, the authors should reflect more on this issue and adjust their methods/interpretations in light of this. One option here would be to extend their discussion of their random forest regression. Indeed, the paper would benefit from extending and deepening the description and results of this analysis, providing results on correlations between nutrient intakes and variance inflation factor. Again, I believe a statistical editor should be consulted.

Thank you for your valuable suggestion for improving the quality of the manuscript. As per academic editor recommendation, we determined to construct a single model by executing the conditional logistic regression. While making a single parsimony model, we excluded one of the variables that showed a multicollinearity problem deciding based on the square root of vif value >2 as a cutup point. However, excluding one of the variables undermines the importance of that for the risk of disease. Therefore, we performed random forest analysis, including all variables in the model, to get the most important variables associated with CAD.

Other data analysis issues:
In Table 1, the authors claim that the median age of authors is statistically significantly different between the case and control groups. The authors report the median age, interquartile range, and P-value as:
Case: 58 (50–65) | Control: 58 (50–65) | P=0.001

Not only is the median age the same, but so too are the interquartile ranges. Yet, despite this, the age difference is apparently statistically significant? The authors seem to interpret this result as meaningful:

“Because the age was matched in five year intervals in the study, the median age was 58 years, which was the same in both case and control groups, respectively, and still showing strong association (P=0.001).”

The authors claim that age was matched in 5-year intervals, but are we then to interpret these results as suggesting that there is actually a significant difference in age between case-control matched pairs? As CAD is strongly associated with age in previous research, this is important to clarify. To do this, the authors need to report the age distribution data for both groups as a supplementary file.

Our study was a matched case-control study, and we exactly matched the “sex," but we matched the “age” at an interval of 5 years. Because of interval matching and Wilcoxon-sign ranked test for paired data, “age” was showing significant association with outcome. Wilcoxon sign-rank sum test for non-parametric paired data and McNemar test for dichotomous paired data are the correct test for the analysis in the matched case-control study [1]. Matching in a case-control study does not control for confounding by the matching factors. A matched design may require controlling for the matching factors in the analysis [4]. Therefore, even though “sex” and “age” were conditioning variables in the study, we adjusted “age” in the model.

For the analysis in Table 2, the authors claim “as most of the study data were not normally distributed, median and interquartile range of nutrients are presented”. The authors should, therefore, include in their supplementary material the actual data underpinning their analysis. At the very least, they must include the mean, range, and SD for each nutrient, so the reader can understand exactly what the distribution of these data actually are. Indeed, the data provided in the paper is insufficient to replicate the necessary results reported, despite the authors declaration.

We followed the standard statistical norms for the presentation of data. We believe that it looks awkward, putting two averages (median and mean) in a single table representing the average of the same variables. As per your suggestion, we have presented the data in the supplementary table that includes mean, range, and SD (S3 Table).

Statistical rhetoric:
The authors highlight a “highly significant” finding (p.9), this language is inappropriate and should be replaced. A result is simply either significant or non-significant and this is determined by whatever threshold of significance the authors deem necessary.

We appreciate your suggestion. We just changed the language as per your suggestion that “highly significant” to only either significant or non-significant.

4. Problems of variable selection and measurement

Nutrient selection:
The authors select 18 nutrients to examine here, but why these specific nutrients are analysed is not adequately justified. Accordingly, the authors should make clearer why these items were selected for analysis.

We calculated daily nutrient intake from Nepalese, commonly consumed food items based on "Nepal food composition table 2017," which lacks the values of all nutrients per 100 gm food items are available for the food items. Although we found literature where these 18 nutrients were mostly related to CAD, the results were inconsistent in the different populations across the world.

In the supplementary file, a list of common foods consumed is provided. This raises further questions about why the authors chose only to analyse the variables they selected in this paper because other variables appear possible to derive from their data. For example, I see no reason why the amount of sugars in the diet couldn’t be calculated from the listed food items, so why isn’t this examined in the paper? Similarly, their decision to use total carbohydrate intake as a variable without breaking this down into refined and complex carbohydrates appears strange and problematic, particular because the authors acknowledge in the paper that there are important differences between these. Why then didn’t the authors calculate these?

Yes, we acknowledged that we did not breakdown the total carbohydrate into "refined" and "complex." We have now mentioned this issue in the limitation section.

In its current state, this Table of food items is both uninformative and misleading. It also raises further questions about how nutrient intakes were measured. Patients were asked about milk consumption, but the milk category does not clarify whether respondents were asked specifically about the amount of full, semi, or skimmed milk consumed, which would be necessary to understand fat content and fatty acid profiles, or whether this was a single category. Further questions about how the quantities of PUFA, MUFA, and SFA were calculated arise in regards to several of the vague categories, such as “vegetable oils”. Accordingly, the authors should include the specific dietary survey actually provided to patients. Furthermore, supplying the average amount of each food consumed by cases and controls for each item would shed more light on dietary habits. As recent research suggests different whole foods might have different effects on lipid profiles and thereby atherosclerosis, these data are important to report. At the very least the authors need to make available the intakes of each nutrient examined in this study per patient.

Regarding milk category, Nepalese in rural area usually have a practice of making butter: heating milk and allow cooling down and extracting the butter before consumption of milk in a village. In city area, the marketable milk is semi skimmed milk. Therefore, we calculated categories of fat based on semi skimmed milk. Regarding vegetable oil, we included nutrients value separately (soybean oil, mustard oil, sunflower oil). We did not calculate effect of the different whole food on the lipid profile of patients.

Other questions that arise are why were PUFA here considered as a single group and not split further into Omega-3 and Omega-6 variants? Why was the intake of trans-fats not measured? Thus, the authors need to revise the manuscript to give the reader a clearer understanding of the theoretical justification for the selection of the variables. As there is a voluminous literature on the relationship between diet and atherosclerosis/CHD/CVD extending back to the early 20th century, there is a wonderfully rich literature to draw from.

We calculated nutrients based on the "Nepal food composition table 2017," which lacks all nutrients value for the given food items. This limitation, we have now mentioned in the limitation of the study.

Self-reported nutritional data:
As all the nutritional data are all self-reported, the authors should include a clearly discussion in regards to their reliability given the known problems with this kind of data. I suspect there is a problem here. From Table 2, it appears that total daily nutritional intake was virtually the same in the two groups – despite the significantly higher incidence of obesity in the control group.

According to your suggestion, we have discussed the problem related to the self-reported diet survey. We did not report a higher incidence of obesity in the control group. I think the word “Normal” was making confusion. Therefore, we change the word “Normal” to "No." Now, we reported the BMI for Asian ≥27.5 kg/m^2^ as obesity. So, now the figures in the Table are different from the previous values.

5. Referencing
In-text references in this paper appear occasionally only loosely related to the claims they are purported to be associated with. For example, reference number 2 is inserted after the following sentence:
“In Nepal, 30% of total death was related to cardiovascular disease (2).”

Yet, reference 2 is a paper by Rankinen et al. (2015), and nowhere in this paper is this claim made. Another reference chosen at random, reference 20, is used to support the authors claim that:
“Besides cholesterol is also an independent risk factor of CAD according to the lipid theory”
The paper referenced nowhere discussed dietary cholesterol. It is a paper examining, as the title suggests, the “Relationships Between Components of Blood Pressure and Cardiovascular Events in Patients with Stable Coronary Artery Disease and Hypertension”. The only mention of cholesterol in this paper is HDL-C and LDL-C – that is, cholesterol bound in particular classes of lipoproteins carried in the blood.

If the authors make the rest of the revisions outlined, I will examine each of the references of this paper. So my recommendation would be to go through each reference and ensure it is relevant to the claim being made. As discussed, the authors also need to ensure they have adequately represented the state of research in relation to their claims. If the article is resubmitted, I'll check each.

We are very regretful to inform that while converting from other reference styles (Vancouver) to PLoS one, all reference lists had been mismatched because of some technical problem. Now, we corrected all citation-related issues in the manuscript.

6. Flawed study design
However, there is one problem that may undermine the point of revising this manuscript. The authors have a case-matched control group – but the control group are not healthy individuals, but patients with other health conditions and cardiovascular symptoms. This is clearly evident by the way the authors chose to enrol patients – all patients were being examined because of suspected coronary problems. This makes it impossible to talk of differences between these groups in terms of risk factors.

For example, looking at Table 1, the ‘control’ group has a significantly higher incidence of obesity and central obesity – but it would be obviously wrong to conclude that obesity and central obesity are protective against cardiovascular disease. Here we’re seeing a stratification of phenotypic characteristics between two different patient groups, and from this we can’t conclude anything at all about risk.

In table 1, we did not report a higher prevalence of obesity and central obesity. The percentage we mentioned were about “normal” and also provided in the foot notes. Now, we realized that the word “Normal” is not appropriate and unclear. Therefore, we changed “Normal” to “No”.

This might also explain the extremely strange finding that the number of hypertensives was roughly the same in both the control and case groups - Control: 143 hypertensives (~46.7%) | Case: 142 hypertensives (~46.4%). As hypertension is one of the key known risk factors in the development of CAD/CVD and extensively supported in the literature, this finding requires a lot more reflection. Why were hypertensives so common in the control group? This control group had apparently no evidence of stenosis – so this seems to be quite an important avenue to explore what went on here.

Although hypertension is one of the critical risk factors in the development of CAD/CVD, the same prevalence was reported because the selection of the control groups was from the outdoor patients of the same hospital where more hypertensive patients had come for their hearts checkup. However, we aimed to determine the nutrients factors, not cardiometabolic factors related to CAD. We believe more the matching of the demographic and cardiometabolic factors more precisely can detect the effect size of nutrients to CAD.

Our control group was those patients who were having either stress test negative or normal angiography. Those patients who were recently diagnosed as hypertension apparently did not show stenosis. Mostly the diagnosed patients were taking medicine to control hypertension and dyslipidemia. Notably, we classified them as hypertensive patients.

Accordingly, this design is inappropriate for the authors stated intention: “The present case-control study was designed to determine the association of dietary nutrients with CAD in the Nepalese population”.

If this study is to be published, the authors need to somehow explain why this control group can be considered representative of a broader population. Later in the paper the authors do highlight the results may have been biased due to “the selection of the control group from the outdoor patients from the same hospital where more hypertensive patients come for their heart check-up”, but this seems to critically undermine the entire results of this study.

Our study was focusing on the dietary nutrients related to CAD. Principally, in the case-control study, the matching of the confounding variables, better can predict the risk posed by the intended risk factors (in our study dietary nutrients). We also believe that almost equal distribution of metabolic risk factors in both cases and controls, the mediation effect posed by these metabolic risk factors (obesity, hypertension, and dyslipidemia) were adjusted.

Thank you very much for the critical appraisal of our manuscript, which has helped improve the manuscript quality, making it publishable in a highly famous journal "PLoS One."

 References

1.	Conway A, Rolley JX, Fulbrook P, Page K, Thompson DR. Improving statistical analysis of matched case-control studies. Research in nursing & health. 2013;36(3):320-4. Epub 2013/02/15. doi: 10.1002/nur.21536. PubMed PMID: 23408517.

2.	Aryal K, Neupane S, Mehata S, Vaidya A, Singh S, Paulin F, et al. Non Communicable Diseases Risk Factors: STEPS Survey Nepal 20132014.

3.	Altman DG. Practical statistics for medical research. London: Chapman & Hall, 1991.

4.	Pearce N. Analysis of matched case-control studies. BMJ (Clinical research ed). 2016;352:i969. Epub 2016/02/27. doi: 10.1136/bmj.i969. PubMed PMID: 26916049; PubMed Central PMCID: PMCPMC4770817 interests and declare the following: none. Provenance and peer review: Not commissioned; externally peer reviewed.

5.	Beunza JJ, Puertas E, García-Ovejero E, Villalba G, Condes E, Koleva G, et al. Comparison of machine learning algorithms for clinical event prediction (risk of coronary heart disease). Journal of biomedical informatics. 2019;97:103257. Epub 2019/08/03. doi: 10.1016/j.jbi.2019.103257. PubMed PMID: 31374261.

6.	Brisimi TS, Xu T, Wang T, Dai W, Adams WG, Paschalidis IC. Predicting Chronic Disease Hospitalizations from Electronic Health Records: An Interpretable Classification Approach. Proceedings of the IEEE Institute of Electrical and Electronics Engineers. 2018;106(4):690-707. Epub 2019/03/20. doi: 10.1109/jproc.2017.2789319. PubMed PMID: 30886441; PubMed Central PMCID: PMCPmc6419763.

7.	Zhang X, Dai Z, Lau EHY, Cui C, Lin H, Qi J, et al. Prevalence of bone mineral density loss and potential risk factors for osteopenia and osteoporosis in rheumatic patients in China: logistic regression and random forest analysis. Annals of translational medicine. 2020;8(5):226. Epub 2020/04/21. doi: 10.21037/atm.2020.01.08. PubMed PMID: 32309373; PubMed Central PMCID: PMCPmc7154412.
