# Supplementary material for: Dietary nutrients of relative importance associated with coronary artery disease: Public health implication from random forest analysis
Source: PLoS One. 2020 Dec 10;15(12):e0243063. doi: 10.1371/journal.pone.0243063 (PMC7728256; doi:10.1371/journal.pone.0243063)
Supplement: Attachment — Submitted filename: Response LetterR2.docx [file pone.0243063.s014.docx]

**Responses to the reviewer’s comments**

Thank you so much for all the comments, which were valuable and encouraging to make quality manuscript publishable in the PLoS One journal. We detail our responses to each of the reviewers’ comments in the order they were raised. We believe that we have improved the methodological details for correct interpretation of the study.

1. Study design and generalizability My primary concern remains the conclusions that the authors draw from their analysis. Previously, I highlighted:

   “The authors have a case-matched control group – but the control group are not healthy individuals, but patients with other health conditions and cardiovascular symptoms. This is clearly evident by the way the authors chose to enrol patients – all patients were being examined because of suspected coronary problems. This makes it impossible to talk of differences between these groups in terms of risk factors. If this study is to be published, the authors need to somehow explain why this control group can be considered representative of a broader population. Later in the paper the authors do highlight the results may have been biased due to “the selection of the control group from the outdoor patients from the same hospital where more hypertensive patients come for their heart check-up”, but this seems to critically undermine the entire results of this study.”

   This remains my position. The authors have not adjusted their conclusions or their interpretation of their study in light of the problems related to their study design. There is no attempt in this study to understand whether the control group is representative of the broader Nepalese population, and there is a major risk of selection bias. This is clearly indicated by the similar number of hypertensive patients in the case and control groups. For example, the major conclusion of this paper remains:

Response: Bias arises in case-control studies not because the cases and controls differ on characteristics other than exposure but because the selected controls do not accurately reflect exposure prevalence in the study base-geographic scope, socioeconomic, and behavioral characteristics of the source population for these cases. Conceptually, the study base is populated by the people at risk of becoming identified as cases in the study if they got the disease during the time in which cases are identified. However, finding the base population in hospital control is pragmatically difficult as the conceptual definition of the study base is unclear. Moreover, the sampling procedure (unbiased sampling) in such a study is not practical. Therefore, we carried out a matched case-control study to overcome the above problems. We understand that obtaining perfectly coherent case and control groups from the same study base guarantees that there will be no additional selection bias introduced in the case–control sampling beyond whatever selection bias may be inherent in the underlying cohort. The failure to do so, however, does not automatically produce selection bias; it just introduces the possibility.

Our study aimed to determine the association of nutritional factors with coronary artery disease (CAD). The distribution of our interest variables (dietary nutrients) in the control group was within the range of findings in a previous study conducted among Nepalese [1]; thus, we believe our control group represents the base/source population. We excluded the participants who adopted dietary modification after being diagnosed with any cardiometabolic risk factors (hypertension, dyslipidemia, diabetes, obesity). The hospital where we carried out the data collection was a cardiac referral center (Only one national cardiac center in Nepal), and people come for their health check-ups from across the country. Furthermore, cardiometabolic characteristics of our control group were somehow similar to the base (source) population except for hypertension [2]. Thus, we believe the control represents the broader Nepalese population. Although controls were selected from the same hospital with other health conditions, the effect of such conditions was adjusted during statistical analysis. Moreover, in a case-control study, we believe more the characteristics (demographic, cardiometabolic, lifestyle-related factors) of cases and controls are matched except study variable (nutritional factors), more accurately we can determine the strength of association of study variables with the disease (CAD).

“Thus, a dietary intervention approach in CVDs is an effective strategy to reduce the public health burden. We conclude that dietary SFA, vitamin A.R.E., dietary total fat and oil, β-carotene, and cholesterol are topmost five essential dietary nutrients associated with CAD in the Nepalese population.”

The findings of this paper cannot be extended to make claims about the relationship between the intake of any dietary nutrient in the wider Nepalese population and their risk of CAD. The findings are at best suggestive of a possible relationship between these nutrients and the development of CAD, but prospective cohort studies and RCTs will need to be performed, as the authors do go on to highlight.

Response: Thank you for your constructive feedback. We are pleased to work with the meticulous reviewer and have the opportunity to enhance our knowledge of science. We agreed with your recommendation that a possible relationship between these nutrients and the development of CAD. We have changed our manuscript as per your suggestion and added the statement as

*“The findings are at best suggestive of a possible relationship between these nutrients and the development of CAD, but prospective cohort studies and RCTs will need to be performed.”*

Further, it is also clear that the case group here differs drastically from the control group in many important aspects. Compared to the controls, the case group has more than double the number of patients with diabetes, double the number obese (BMI) patients, triple the number of patients suffering from dyslipidaemia, and triple the number who are current smokers. They cases also drink more alcohol and exercise less than the control group. These groups are not comparable – and clearly have very different lifestyles, so it is not clear to me that the authors can draw any conclusions about the respective role of specific nutrients in explaining CAD between the groups.

Response: If the comparison was performed only each nutrient with CAD without adjustment with the above-mentioned variables like in 2*2 table, these groups were not comparable. However, our conclusion was based on random forest analysis and multivariable conditional logistic regression, where the effects of these confounding variables were adjusted. Therefore, conclusions about the respective role of specific nutrients in CAD between the groups were justifiable.

Accordingly, more needs to be done to modify the conclusions of this paper and highlight the limitations of this study before publication. As there are questions over the generalisability of these findings beyond the study population, my recommendation would be to limit all conclusions to describing the findings of this study in relation to this group alone.

Response: We agreed with your recommendation to limit all conclusions to describing the findings of this study about this group alone, and have changed our manuscript accordingly. The revised sentences were as:

*“Our study suggests higher dietary intake of β-carotene and vitamin C are possible protective dietary nutrients, while an increased intake of dietary SFA, total fat and oil, and cholesterol are potential risk factors for CAD development. However, prospective cohort and RCTs studies with a large sample size are needed to explore the causal link of these nutrients for the risk of CAD development in the Nepalese population.”*

2. Unexplained differences in findings reported between the original and revision.
   The authors must explain why some figures in this revised manuscript compared to the original have changed. Specifically, I refer to Table 2. In the original, for Food energy (kcal) per day, the controls are reported as 2560 (2306, 2791) and the cases 2549 (2256, 2897) kcal. However, in the revised manuscript, the controls now are reported as 2674 (2445, 2909) and the cases 2622 (2373, 2963). Despite this change, none of the other macronutrient values have changed, which cannot be true.

   Further, I cannot understand how the authors have also changed values for PUFA and SFA intake in this revised version compared to the original – but somehow this has not changed the value for total fat intake?
   In this original paper,
   Fat g Control 56 (47, 64) | Case 61 (52, 72)
   PUFA g Control 18 (11.2, 22.7) | Case 19.6 (11.8, 25.7)
   SFA g Control 15.8 (10.8, 20)| Case 16.6 (11, 21.6)

   In the revised manuscript,
   Total fat/oil g Control 56 (47, 64)| Case 61 (52, 72)
   PUFA g Control 18.7 (12.5, 23.5) | Case 19.6 (12.4, 25.7)
   SFA g Control 15.5 (10.6, 19.2) | Case 19 (13.9, 23.6)

   The authors need to explain why this discrepancy has occurred because it undermines my confidence in the reliability of these data. I am particularly worried about the change in SFA values because the authors now report a significant OR of 1.2 (1.11, 1.31) for SFA intake in the revised manuscript, which was not included in the original.

Response: We apologize that we did not explain why some figures in the revised manuscript compared to the original had changed in the previous review. We calculated the nutrients of individual observation from the nutrients calculator developed in an excel sheet based on the value from Nepal food composition table 2017 and transferred to the datasheet. We meticulously re-check our nutrients conversion from the food list. While transferring the data, some values were missing in SFA. Therefore value in the SFA has been changed while total fat/oil intake remains the same. The same problems happened to data for food energy per day.

Another major change is the figures concerning vitamin A,E,R intake between these versions:
In the original:
Vitamin A R.E. Control 739 (578, 885)| Case 657 (535, 790)
In the revised:
Vitamin A R.E. Control 698 (546, 836)| Case 622 (506, 728)
Either there was a major problem with the basic statistical analysis performed for the previous version of this paper, or these data have since been changed.

Response: Regarding Vitamin A.R.E, the value of Vitamin A.R.E per 100g vegetables was incorrectly placed in the nutrient calculator formula. After correcting this problem, the final value has been changed.

3. Referencing problems
   Some references still do not clearly relate to the statements made by the authors. For example, the authors state:
   "An estimated 7.4 million people died from CAD in 2015, representing 13% of all global deaths. In Nepal, 30% of total death was related to cardiovascular disease [2]".

   The reference provided for this is “WHO. Cardiovascular diseases fact sheet. WHO. World Health Organization; 2017”. Nowhere in this referenced document are either of these figures provided.

Response: We have cited it from the WHO webpage and changed to the correct citation for that webpage as [3]. Also we removed the statement, “In Nepal, 30% of total death was related to cardiovascular disease” from that sentence in the manuscript.

4. Summary
   My recommendation, despite the improvements to the paper in many areas, remains to reject this paper. Previously, this was based on the flawed study design that, I believed, undermined the generalisability of these results to the broader Nepalese population. However, the unexplained changes to nutrient intakes in this revised version undermined my confidence in this study.

Response: We have addressed the issue raised and revised our manuscript as per reviewer’s suggestions regarding the generalizability of the results. We have meticulously re-checked each nutrient’s value so that current values differ from the original manuscript (first version). We again apologize for the reason for changes that were not mentioned in the previous review. Finally, we have changed the manuscript as per your advice (an expert reviewer) that we believe enough quality for publication in the current journal.

References

1.	Shrestha A, Koju RP, Beresford SAA, Chan KCG, Connell FA, Karmacharya BM, et al. Reproducibility and relative validity of food group intake in a food frequency questionnaire developed for Nepalese diet. International journal of food sciences and nutrition. 2017;68(5):605-12. Epub 2017/01/18. doi: 10.1080/09637486.2016.1268099. PubMed PMID: 28092991.

2.	NHRC. Nepal Steps Survey 2019-Facts Sheets-Nepal Health Research Council. [retrieved 2020-10-06]; Available from: <http://nhrc.gov.np/nepal-steps-survey-2019-fact-sheets/>.

3.	WHO. Cardiovascular diseases fact sheet. World Health Organization; 2017. [retrieved 2020-7-30]; Available from: https://[www.who.int/en/news-room/fact-sheets/detail/cardiovascular-diseases-(cvds)](http://www.who.int/en/news-room/fact-sheets/detail/cardiovascular-diseases-(cvds)).
